# Human umbilical cord mesenchymal stromal cell small extracellular vesicle transfer of microRNA-223-3p to lung epithelial cells attenuates inflammation in acute lung injury in mice

**DOI:** 10.1186/s12951-023-02038-3

**Published:** 2023-08-25

**Authors:** Jie Chen, Shiyang Ma, Baihua Luo, Haojie Hao, Yanqin Li, Hang Yang, Fei Zhu, Peipei Zhang, Ruichao Niu, Pinhua Pan

**Affiliations:** 1 Department of Respiratory Medicine, Clinical Research Center for Respiratory Disease, Xiangya Hospital, National Key Clinical Specialty, Branch of National, Central South University, No.28 Xiangya Road, Kai-Fu District, Changsha, 410008 Hunan China; 2 Center of Respiratory Medicine, Xiangya Hospital, Central South University, Changsha, 410008 Hunan China; 3 Clinical Research Center for Respiratory Diseases in Hunan Province, Changsha, 410008 Hunan China; 4 Hunan Engineering Research Center for Intelligent Diagnosis and Treatment of Respiratory Disease, Chang-sha, 410008 Hunan China; 5 National Clinical Research Center for Geriatric Disorders, Xiangya Hospital, Changsha, 410008 Hunan P.R. China; 6 Department of Pathology, Xiangya Hospital, Central South University, Changsha, Hunan Province China; 7 Institute of Basic Medicine Science, Chinese People’s Liberation Army General Hospital, Chinese People’s Liberation Army Medical College, Beijing, China; 8 Center of Pulmonary & Critical Care Medicine, Chinese People’s Liberation Army (PLA) General Hospital, Chinese PLA Medical College, Beijing, China; 9 Department of Respiratory Medicine, The Second Affiliated Hospital of Xinjiang Medical University, Xinjiang Uygur Autonomous Region, Urumqi, China

**Keywords:** Mesenchymal stromal cells, Small extracellular vesicles, Acute lung injury, MicroRNA-223-3p, Inflammation

## Abstract

**Background:**

Acute lung injury (ALI), manifested as strong pulmonary inflammation and alveolar epithelial damage, is a life-threatening disease with high morbidity and mortality. Small extracellular vesicles (sEVs), secreted by multiple types of cells, are critical cellular communication mediators and can inhibit inflammation by transferring bioactive molecules, such as microRNAs (miRNAs). Thus, we hypothesized that sEVs derived from mesenchymal stromal cells (MSC sEVs) could transfer miRNAs to attenuate inflammation of lung epithelial cells during ALI.

**Methods:**

C57BL/6 male mice were intratracheally administered LPS (10 mg/kg). Six hours later, the mice were randomly administered with MSC sEVs (40 µg per mouse in 150 µl of saline), which were collected by ultracentrifugation. Control group received saline administration. After 48 h, the mice were sacrificed to evaluate pulmonary microvascular permeability and inflammatory responses. *In vitro*, A549 cells and primary human small airway epithelial cells (SAECs) were stimulated with LPS with or without MSC sEVs treatment.

**Results:**

*In vitro*, MSC sEVs could also inhibit the inflammation induced by LPS in A549 cells and SAECs (reducing TNF-α, IL-1β, IL-6 and MCP-1). Moreover, MSC sEV treatment improved the survival rate, alleviated pulmonary microvascular permeability, and inhibited proinflammatory responses (reducing TNF-α, IL-1β, IL-6 and JE-1) in ALI mice. Notably, miR-223-3p was found to be served as a critical mediator in MSC sEV-induced regulatory effects through inhibition of poly (adenosine diphosphate-ribose) polymerase-1 (PARP-1) in lung epithelial cells.

**Conclusions:**

Overall, these findings suggest that MSC sEVs may offer a novel promising strategy for ALI.

**Supplementary Information:**

The online version contains supplementary material available at 10.1186/s12951-023-02038-3.

## Introduction

Acute lung injury (ALI) manifests as acute respiratory distress syndrome (ARDS) in patients [1]. ALI/ARDS is a life-threatening condition characterized by diffuse interstitial and alveolar edema and widespread inflammation in the lung [2–4]. Damage to alveolar epithelial cells (AECs) plays an essential role in the progression of ALI [5, 6]. Despite substantial progress in medical therapies and ventilation management, the mortality of ARDS remains as high as 40% [4]. Therefore, novel therapeutic strategies that target specific pathologies to inhibit the development of ALI/ARDS are urgently needed.

Mesenchymal stromal cells (MSCs) have been demonstrated to hold great potential to promote inflammation resolution and tissue repair in many kinds of ALI models (endotoxin [7, 8], sepsis [9], and live bacteria [10]). Moreover, MSC-based treatment has shown promising results in ARDS clinical trials [11, 12]. However, the limitations and challenges of MSC therapy, such as the low survival rate in vivo and potential immune rejection and tumor generation, should not be ignored [13, 14]. In the past few years, we and others have reported that MSC-conditioned medium has the same therapeutic effect as MSCs in both in vitro and in vivo models of ALI [15–17]. Recently, mounting evidence has confirmed that the main mechanism of MSCs achieving their restorative properties in ALI is a paracrine mechanism instead of cell engraftment [18, 19].

Extracellular vesicles (EVs), secreted by MSCs through paracrine signaling, have been demonstrated to be a critical cell-free approach for the therapy of multiple pathological diseases [20–23]. EVs are nanosized, lipid bilayer-enclosed particles that play an important role in intercellular communication [24]. Based on the guidelines of the Minimal Information for Studies of Extracellular Vesicles, EVs are mainly categorized as either medium-to-large EVs (ranging from 150 to 1,000 nm) or small EVs (sEVs) (~ 30–150 nm) according to their size and mechanisms of biosynthesis [25]. EVs have been demonstrated to be involved in intercellular communication mainly by transferring proteins, lipids, and genetic materials (mRNA, ncRNA, etc.) between the source and target cells [24]. Distinct surface ligands present on the surface of sEVs ensure their cell type–specific and organotropic targeting, thereby regulating specific biological functions [26]. Transplantation of MSC sEVs not only shows similar biological influences as direct transplantation of MSCs but also has the advantages of targeted delivery, low immunogenicity, and high repairability [14, 27]. Currently, numerous studies have reported the beneficial effects of MSC-derived EVs in tissue engineering, inflammation modulation, regeneration medicine, etc. [28–30]. Our prior work also showed that sEVs released from MSCs inhibited the inflammatory response, reduced pulmonary edema, and improved survival in a sepsis-induced ALI mouse model [31]. Importantly, sEVs derived from MSCs have been reported to be successfully treated in patients with graft-versus-host disease (GvHD) [32]. Recent clinical trials have also been carried out with the purpose of evaluating the effects of MSC-derived sEVs on the therapy of several diseases [33].

A growing number of studies have confirmed the critical role of MSC sEVs in improving the pathology of ALI. The underlying mechanisms have been demonstrated to be related to the sEV content, including proteins, mRNAs and miRNAs [34–36]. However, the mechanisms remain incompletely understood. Here, we observed that human MSC-derived sEVs could be taken up by AECs in vitro. sEVs released by MSCs could inhibit inflammation both in vivo and in vitro in an ALI model. Moreover, we found that the levels of miR-223-3p were highly elevated in MSC sEVs and that poly (adenosine diphosphate-ribose) polymerase–1 (PARP-1) functioned as the downstream target of sEV miR-223-3p in modulating inflammation. Mechanistically, miR-223-3p overexpression or inhibition in MSC sEVs impaired or enhanced the inflammatory response in ALI.

## Materials and methods

### Cell culture and treatment

Our study was approved by the human ethics committee of the Chinese People’s Liberation Army (PLA) General Hospital. Umbilical cords (UCs) were donated by six healthy babies whose parents signed the informed consent form. Human umbilical cord Wharton’s jelly-derived MSCs (hUC-MSCs) were extracted and cultured as described previously [31, 37]. In brief, UCs were washed with phosphate-buffered saline (PBS) three times. After removing arteries, veins and amnion, UCs were cut into 1.0- to 2.0-cm segments. Wharton’s jelly was separated and minced into 1 mm^3^ pieces and then cultured in Dulbecco’s modified Eagle’s medium (DMEM) with 10% fetal bovine serum (FBS). When hUC-MSCs were at passage six and grew to 80% confluence, their medium was replaced with serum-free medium (Cyagen Biosciences Inc., Guangzhou, China).

The A549 cell line was purchased from the Chinese Academy of Science (Shanghai, China) and cultured in DMEM with 10% FBS. These cells were identified by the marker for alveolar type II epithelial cells with an immunofluorescence assay in our previous publication [15].

Human primary small airway epithelial cells (SAECs) were procured from ScienCell Research Laboratories (San Diego, CA, USA) and cultured in SAEC medium (Cat. # 3231, ScienCell) according to the instructions.

MiR-223-3p inhibitors (50 nM) and their negative controls (50 nM) QIAGEN, Germantown, MD, USA) were transfected into hUC-MSCs (1 × 10^5^ cells for each 12-well) by HiPerFect transfection reagent (QIAGEN, Germantown, MD, USA) based on the presented instructions. The sequences were as follows: miR-223-3p Inhibitor: 5′-UGGGGUAUUUGACAAACUGACA-3′, negative control of inhibitor: 5′-CAGUACUUUUGUGUAGUACAA-3′. After 6 h of transfection, the culture medium was changed to serum-free medium (Cyagen Biosciences Inc., Guangzhou, China). After 42 h, the sEVs were extracted according to the differential ultracentrifugation method. The collected sEVs were used for the subsequent experiments.

For the inhibition of PARP-1 expression, A549 cells or SAECs were transfected with PARP-1 small interfering RNAs (siRNAs) (siPARP-1 #1, 2 and 3) or universal negative control siRNA (siCtrl). The primers were as follows: siPARP-1 #1: 5′-CGGATAAGCTCTATCGAGTCGAGTA-3′, siPARP-1 #2: 5′- GGAAGTGAAAGCAGCCAAT-3′, siPARP-1 #3: 5′- CGAGAAATCTCTTACCTCAAGAAAT-3′, siCtrl: 5′- GGCTCCGATCGTCTCACAT-3′.

### sEVs isolation, characterization, and treatment

MSC sEVs were obtained from the supernatant of serum-free medium-cultured hUC-MSCs with a differential ultracentrifugation method. In brief, the supernatant was centrifuged at 300 × g for 10 min and 2000 × g for 10 min. Then the supernatant was ultracentrifuged 10 000 × g for 30 min twice. After those centrifugations to remove dead cells, membranes and cell debris, the supernatant was filtered through a 0.22 μm filter. Then, the supernatant underwent ultracentrifugation, and the pellets containing sEVs were collected and resuspended in PBS for storage. The details are described in our previous publication [31].

To characterize the separated sEVs, their surface biosignature proteins, including CD81, TSG101, and calnexin, were observed by Western blotting. To detect the size distribution and concentration of the sEVs, tunable resistive pulse sensing (TRPS, qNano, Izon Science) analysis was performed. To identify the morphology of the sEVs, transmission electron microscopy (TEM, Hitachi H-7650, Japan) was performed. The protein concentrations of sEVs were detected using a bicinchoninic acid (BCA) protein assay kit (Pierce Chemical, Rockford, IL, USA). All the details were shown in Figure S1 and Table S2 and also in our recent publication [31].

For the in vivo experiment, MSC sEVs were injected into ALI or sham mice through the tail vein (40 µg per mouse in 150 µl of saline). For the in vitro experiment, MSC sEVs were administered to the culture medium of A549 or SAECs (2 µg per 1 × 10^5^ in 10 µl of PBS). To determine whether MSC sEVs can be taken up by epithelial cells, sEVs were marked with a green fluorescent dye (PKH67; Sigma).

To trace the administered MSC sEVs in vivo, Dil (D282, Thermofisher Scientific) labeled sEVs (40 µg per mouse in 150 µl of saline) were injected intravenously into mice. Then fixed frozen sections of mouse lung were incubated overnight at 4 °C with primary antibody of podoplanin (ab256559, 1:500; Abcam, Cambridge, UK). The slides were incubated with fluorescently conjugated secondary antibodies for 30 min at room temperature in the dark. DAPI was used for nuclear staining.

Fluorescence images were captured using an Olympus IX81 microscope (Olympus, Tokyo, Japan) with an attached CoolSNAP HQ2 CCD camera (Teledyne Photometrics, Arizona, USA) and a 20X or 40X objective lens (Olympus, Tokyo, Japan). Different fluorescence was detected with different excitation and emission bandpass filters (DAPI (Chroma Cat. #: 31000v2): excitation / emission = 350(50) nm / 460(50) nm for bule fluorescence; Endow GFP (Chroma Cat. #: 41,017): excitation / emission = 470(40) nm / 525(50) nm for green fluorescence; Texas Red (Chroma Cat. #: 41,004): excitation / emission = 560(55) nm / 645(75) nm for red fluorescence). Samples were imaged at magnifications of 1.6X using a 0.38 or 0.62 μm z-step.

To quantify the retention of MSC sEVs, the number of nuclei of podoplanin positive cells surrounded with Dil-labeled MSC sEVs was divided by the total amount of nuclei of podoplanin positive cells. Five random tissue sections were selected per mice. The positive cells were calculated with ImageJ (https://imagej.nih.gov/ij/).

### Coculture experiments

**Fig. 1:**
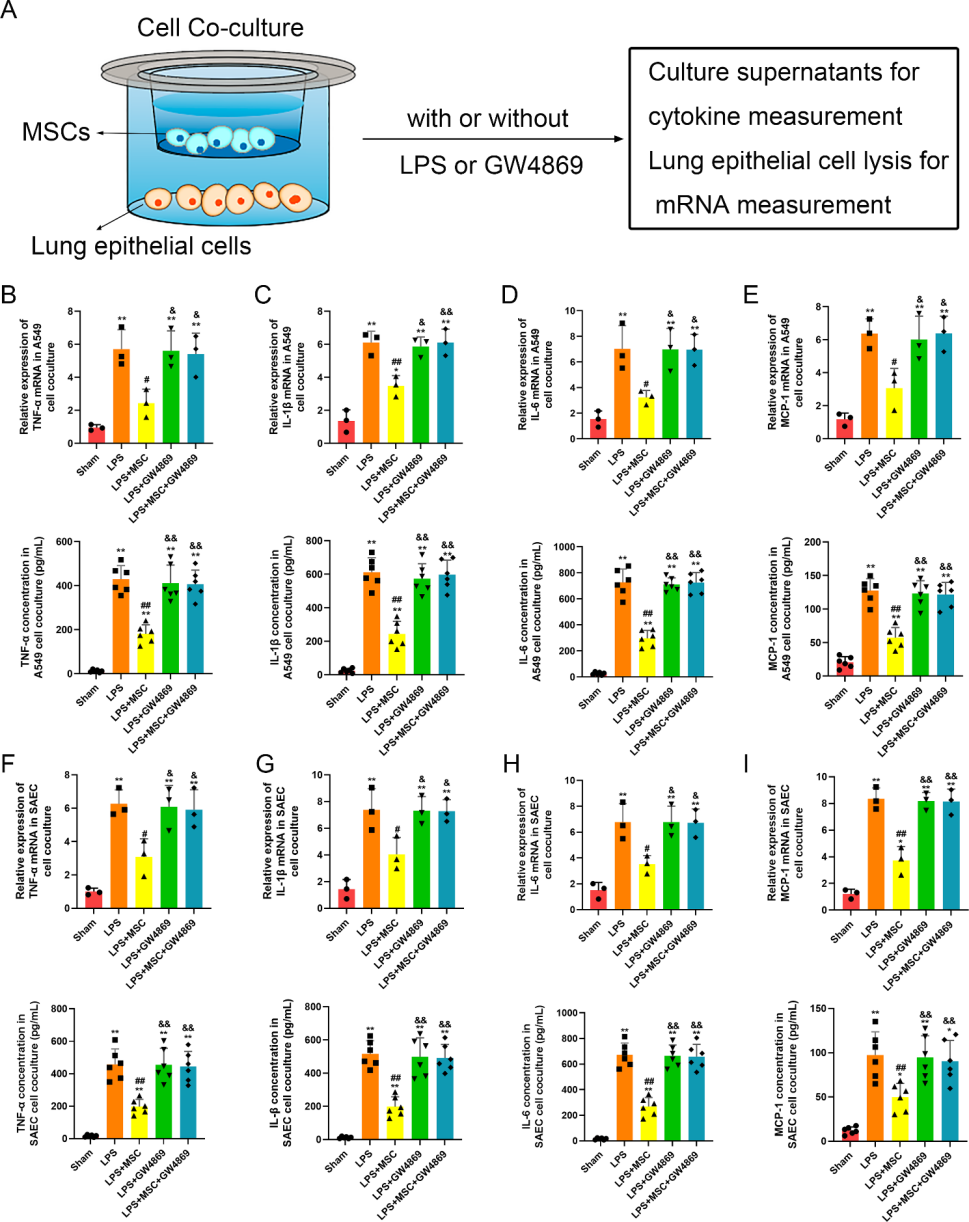
Mesenchymal stromal cells (MSCs) suppressed LPS-induced inflammation in lung epithelial cells through small extracellular vesicles (sEVs) (**A**) A diagram of cell co-culture system in which A549 cells or small airway epithelial cells (SAECs) were cultured in the lower chamber and MSCs were cultured in the upper chamber of a 12-well insert (**B-E**) mRNA and protein levels of (**B**) tumor necrosis factor alpha (TNF-α), (**C**) interleukin-1β (IL-1β), (**D**) interleukin-6 (IL-6), and chemokines (**E**) macrophage chemoattractant protein-1 (MCP-1) in A549 cells which were co-cultured with or without MSCs. (**F-I**) mRNA and protein levels of (**F**) tumor necrosis factor alpha (TNF-α), (**G**) interleukin-1β (IL-1β), (**H**) interleukin-6 (IL-6), and chemokines (**I**) macrophage chemoattractant protein-1 (MCP-1) in SAECs which were co-cultured with or without MSCs. Statistical analysis: one-way ANOVA with a Tukey-Kramer post hoc test. * *P* < 0.05, ** *P* < 0.01, compared between the Sham group and each treated group (LPS, LPS + MSC, LPS + GW4869, LPS + MSC + GW4869). # *P* < 0.05, ## *P* < 0.01, compared between the LPS and the treated group (LPS + MSC, LPS + GW4869, LPS + MSC + GW4869). & *P* < 0.05, && *P* < 0.01, compared between the LPS + MSC and the LPS + GW4869 or LPS + MSC + GW4869 group. n = 3 for mRNA and n = 6 for protein in each group

Cell coculture was conducted on Transwell plates with a 0.4 μm pore size membrane (6-well or 12-well insert, Corning Inc.). Epithelial cells (A549 and SAECs) were seeded in the lower chamber (1 × 10^5^ cells for each 12-well, 2 × 10^5^ cells for each 6-well), while MSCs were seeded in the upper chamber (1 × 10^5^ cells / 12-well, 2 × 10^5^ cells / 6-well). LPS from Escherichia coli O55:B5 (E. coli 055:B5; Sigma) (100 ng/mL) was added to the lower chamber, while GW-4869 (20 µM, Sigma-Aldrich) (blocking the release of sEVs) was added to the upper chamber according to the study design (Fig. 1A).

### Animals and treatment

All animal experiments were approved by the Institutional Animal Care and Ethics Committee of Xiangya Hospital. C57BL/6 male mice weighing 18–22 g were maintained in a germ-free environment (temperature, 22 ± 2 °C; humidity, 55 ± 5%). The total number of the mice used in this study was three hundred and thirty-five. All the mice were obtained from the Laboratory Animal Center, Xiangya School of Medicine, Central South University (Changsha, China). Firstly, one hundred and twenty mice were randomly divided into four groups. After anaesthetization with pentobarbital (90 mg/kg), the mice were intratracheally administered LPS (10 mg/kg) (sixty mice) or the same dose of saline as a control (sixty mice) [8] (Figure S2 and S3). Six hours later, the control or LPS mice were randomly administered saline or MSC sEVs (40 µg per mouse in 150 µl of saline) through the tail vein. To prevent the effect of bronchoalveolar lavage (BAL) on lung tissue analysis, each group of mice was divided into two subgroups: one subgroup was used only for collecting BAL fluid (BALF), while the other was used for collecting lung tissue samples (n = 15 for each subgroup) [8] (Figure S4). Secondly, to explore the underlying mechanisms of MSC sEVs on ALI, seventy-five mice were randomly divided into five groups: sham, LPS + PBS, LPS + MSC sEVs, LPS + sEVs(223 inhibitor), and LPS + sEVs(ctrl inhibitor) (15 for each group). After 48 h of LPS administration, all the mice were euthanized with pentobarbital sodium (150 mg/kg ip, induction of unconsciousness) to achieve a peaceful and painless death.

To evaluate the survival rate, firstly, an additional sixty mice were randomly divided into three groups (LPS + PBS, Sham + MSC sEVs, LPS + MSC sEVs). Secondly, an additional eighty mice were randomly divided into four groups (LPS + PBS, LPS + MSC sEVs, LPS + MSC sEVs (miR-223-3p inhibitor), and LPS + MSC sEVs (control inhibitor)) to explore the underlying mechanisms of MSC sEVs on ALI. The rectal temperature of the mice was measured every 12 h until 168 h. When their rectal temperature was below 30 °C, the mice were considered nearly dead and sacrificed humanely (intraperitoneal injection of pentobarbital sodium 150 mg/kg) according to our previous publication [8]. The details are shown in supplementary files 1.

### Lung tissue analysis

The pulmonary circulation was flushed with 1 mL cold (4 °C) PBS via the right ventricle injection to remove blood-borne elements and plasma. The right lower lobe of the lung was fixed in 4% paraformaldehyde for 24 h. The lobes were sliced into 4–6 μm sections after embedding in paraffin. After staining with hematoxylin and eosin (HE), the sections were observed under an Eclipse E800 microscope (Nikon, Tokyo, Japan). The images were evaluated by an investigator who was blinded to the identity of the slides. Lung injury scores were assessed by five categories, including edema, hemorrhage, neutrophil infiltration, thickness of the alveolar wall and formation of hyaline membrane. Each category was graded according to a five-point scale [38].

The left lungs of mice were immediately removed after the experiment and dried in a 60 °C oven for two days. The lung wet/dry ratio was calculated to assess lung edema [8]. The right upper and middle lobes of lung tissues were stored at -80 °C for biochemical analysis.

BAL was performed by infusing 1 mL of cold saline into the lung through tracheal intubation three times, and almost 90% of BALF could be recovered [8]. The collected BALF was centrifuged at 500 × g for 10 min at 4 °C. The supernatants were stored at 4 °C for the subsequent experiment. The cell pellet was resuspended with 1 mL of saline. One hundred microliters of this cell suspension was transferred to a glass slide, stained with Wright-Giemsa dye and observed under an Eclipse E800 microscope (Nikon, Tokyo, Japan).

### Small RNA sequencing

The total RNAs of MSC and MSC sEVs were extracted and used for miRNA sequencing (miRNA-seq). Library preparation and miRNA-seq were performed by Beijing Genomics Institute. The PCR products were sequenced using the HiSeq 2500 platform (Illumina, San Diego, CA, USA).

### Quantitative real-time polymerase chain reaction (qRT-PCR)

Total RNA was isolated using TRIzol Reagent (GibcoBRL, Life Technologies) (for tissues and cells) or the SeraMir Exosome RNA Purification Kit (System Biosciences, Mountain View, USA) (for MSC sEVs). RNA concentrations and quality were tested by a Nanodrop 2000 (Thermo Fisher Scientific), and 1 µg of RNA was reverse transcribed into cDNA using a TaqMan microRNA assay kit (QIAGEN, Germantown, MD, USA). Then, 2 µl of cDNA was used for qRT-PCR amplification by SYBR Green Master Mix (Takara Biomedical Technology (Beijing) Co., Beijing, China). Glyceraldehyde 3-phosphate dehydrogenase (known as GAPDH) or U6 was used as the endogenous control gene. The 2-ΔΔCt comparative method was used to calculate the fold-change in gene expression. The specific primer sequences are shown in Table S1.

### Reporter vector construction and dual-luciferase reporter assay

Sequences corresponding to the 3′-UTR of PARP-1 mRNA and containing the predicted miR-223-3p binding sites were synthesized and then cloned into a pGL3 luciferase control reporter vector (Promega, Madison, WI, USA). The PARP-1 3ʹ-UTR reporter constructs (pGL3-WT-PARP-1 and pGL3-MUT-PARP-1) or NFIA-1 3ʹ-UTR reporter constructs (pGL3-WT-NFIA and pGL3-MUT-NFIA) and miR-223-3p mimic or mimic-NC were transfected into 293T cells. Twenty-four hours later, the Dual-Luciferase Reporter Kit (Promega, Madison, WI, USA) was used to measure the luciferase activity.

### RNA-pull down

Probes, including biotinylated miR-223-3p mimic, biotinylated miR-223-3p Mut, and control probes purchased from RiboBio (Guangzhou, China) purchased from RiboBio (Guangzhou, China) were transfected into A549 and SAECs cells. Then DNaseI was added to RNA solution, and incubation was for 5 min at 65 °C. Afterwards, streptavidin-coated magnetic beads (Invitrogen, Carlsbad, CA, USA) were incubated for 4 h. qRT-qPCR was carried out to analyze the enrichment of the co-deposited PARP-1 RNA.

### ELISA analysis and myeloperoxidase (MPO) activity

Levels of tumor necrosis factor alpha (TNF-α), interleukin-1β (IL-1β), interleukin-6 (IL-6), and macrophage chemoattractant protein-1 (MCP-1, called JE-1 in mice) in BALF and cell supernatant samples were determined by cytokine and chemokine enzyme-linked immunosorbent assays (ELISAs) (R&D Systems) according to the manufacturer’s protocol. The MPO activity in the lung tissue was determined by an MPO assay kit (Nanjing Jiancheng Corp., Nanjing, China) according to the details described in our recent publication [31].

### Western blotting analysis

For Western blotting experiments, cells or tissues were incubated in RIPA buffer (Millipore, Temecula, CA, USA) with protease inhibitors for lysis. Protein samples (40 µg) were mixed with 6× loading dye, subjected to 10% SDS-PAGE, and electrotransferred onto PVDF membranes (Millipore, Billerica, MA, USA). Blocking was performed for one hour at room temperature with 5% (w/v) nonfat dry milk. The membranes were incubated with primary antibodies against PARP-1 (#sc-136,208, 1:200; Santa Cruz Biotechnology, USA), CD81 (ab79559, 1:300; Abcam, Cambridge,

UK), TSG101(ab125011,1:1000; Abcam, Cambridge, UK), calnexin (sc23954, 1:200; Santa Cruz Biotechnology, USA) and β-actin (#3700, 1:1,000, CST, USA) overnight at 4 °C on a rotator. Subsequently, blots were incubated with HRP-conjugated secondary antibody for two hours and detected by an enhanced chemiluminescence (ECL) kit.

### Statistical analysis

The quantitative statistics are expressed as the mean ± standard deviation (SD). One-way analysis of variance (ANOVA) was used to determine the significance of differences between the groups. Survival curves were compared by means of the Kaplan-Meier estimator, and significant differences were identified by the log-rank test. A *P* value of < 0.05 was considered statistically significant. All statistical analyses were performed with GraphPad Prism 9.0. (GraphPad Software, La Jolla, CA, USA).

## Results

### MSCs suppressed LPS-induced inflammation in lung epithelial cells

MSCs and A549 cells were characterized in our previous publication [15]. To analyze the effect of MSCs on LPS-induced inflammation in lung epithelial cells, we employed an in vitro coculture system of A549 cells or SAECs with MSCs, where both cell types were separated by a membrane to prevent direct cell-cell contact (Fig. 1A). We observed that LPS remarkably stimulated proinflammatory cytokines (TNF-a, IL-1β and IL-6) and the chemokine MCP-1 in A549 (Fig. 1B-E) and SAECs (Fig. 1F-I) at both the mRNA and protein levels. The proinflammatory cytokines (TNF-a, IL-1β and IL-6) and chemokine MCP-1 in A549 cells and SAECs upon LPS challenge were dramatically reduced by coculture with MSCs (Fig. 1B-I). However, this suppression was reversed by pretreatment with GW4869, which is an inhibitor of the release of sEVs (Fig. 1B-I). These results confirmed that MSCs inhibit LPS-induced inflammation in lung epithelial cells, which may be related to their release of sEVs.

### MSC sEVs suppressed LPS-induced inflammation in lung epithelial cells

**Fig. 2:**
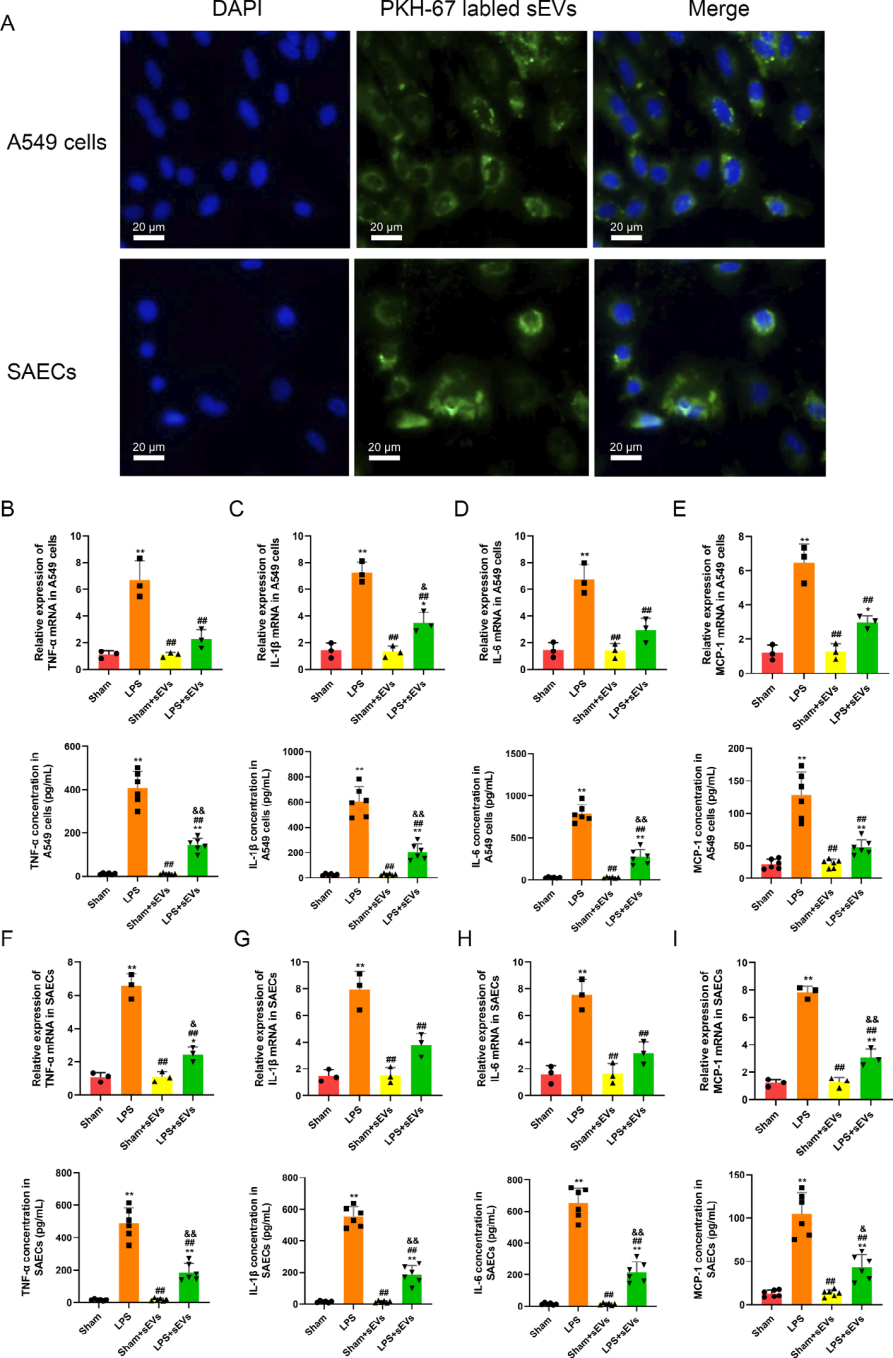
MSC small extracellular vesicles (sEVs) suppressed LPS-induced inflammation in lung epithelial cells (**A**) Fluorescence microscopy analysis of PKH67-labeled MSC sEVs internalization by A549 cells and SAECs. The green labeled MSC sEVs were visible in the perinuclear region of recipient cells. Scale bar = 20 μm (**B-E**) mRNA and protein levels of (**B**) tumor necrosis factor alpha (TNF-α), (**C**) interleukin-1β (IL-1β), (**D**) interleukin-6 (IL-6), and chemokines (**E**) macrophage chemoattractant protein-1 (MCP-1) in A549 cells which were treated with or without MSC sEVs (**F-I**) mRNA and protein levels of (**F**) tumor necrosis factor alpha (TNF-α), (**G**) interleukin-1β (IL-1β), (**H**) interleukin-6 (IL-6), and chemokines (**I**) macrophage chemoattractant protein-1 (MCP-1) in SAECs which were treated with MSC sEVs Statistical analysis: one-way ANOVA with a Tukey-Kramer post hoc test. * *P* < 0.05, ** *P* < 0.01, compared between the Sham group and each treated group (LPS, Sham + sEVs, LPS + sEVs). # *P* < 0.05, ## *P* < 0.01, compared between the LPS and the treated group (Sham + sEVs, LPS + sEVs). & *P* < 0.05, && *P* < 0.01, compared between the Sham + sEVs and the LPS + sEVs group. n = 3 for mRNA and n = 6 for protein in each group

sEVs derived from hUC-MSCs were identified and characterized in Figure S1 and also in our recent publication [31]. We also tested sEV protein content and the results were shown in Table S2. To determine whether MSC sEVs could be internalized into A549 cells and SAECs, MSC sEVs were labeled with PKH67 (green) and then treated in LPS-challenged A549 cells or SAECs for six hours. We found that PKH67 (green)-labeled MSC sEVs were internalized by A549 cells or SAECs (Fig. 2A, Figure S5). Furthermore, we observed that MSC sEVs were able to inhibit proinflammatory cytokines (TNF-a, IL-1β and IL-6) and the chemokine MCP-1 in both LPS-challenged A549 cells (Fig. 2B-E) and SAECs (Fig. 2F-I). These results provide further evidence that the MSC-induced positive effects on LPS-stimulated lung epithelial cells may occur through sEVs.

### MSC sEV treatment improved survival and attenuated lung injury in LPS-induced ALI mice

**Fig. 3:**
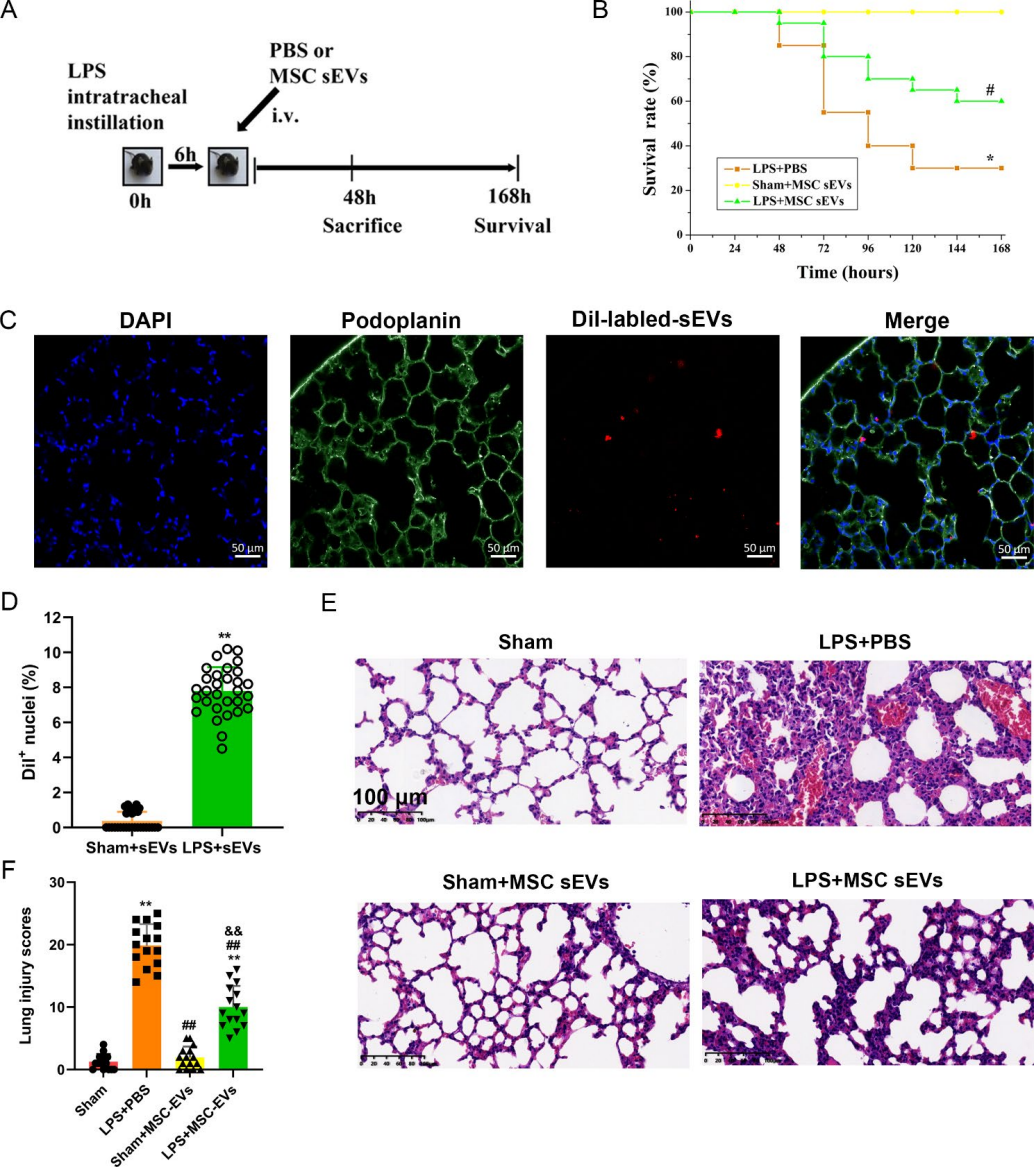
MSC small extracellular vesicles (sEVs) improved survival and attenuated lung injury in LPS-induced ALI mice (**A**) Experimental design for the in vivo study. Mice were intratracheally administered with LPS (10 mg/kg) or Saline. PBS or MSC sEVs (40 µg per mouse in 150 µl of PBS) was injected through the tail vein 6 h after LPS administration. Mice were then sacrificed 48 h to evaluate the therapeutic efficacy or observed until 168 h to evaluate the survival rate (**B**) Kaplan-Meier survival curves showed that administration of MSC sEVs significantly improved survival at 168 h in LPS-induced ALI mice (n = 20 mice per group, *P < 0.05, compared between the Sham + sEVs and the LPS + PBS group. # *P* < 0.05, compared between the LPS + PBS and the LPS + MSC sEVs group (**C**) Representative images of MSC sEVs incorporation in lung epithelial cells in lung tissues. Scale bar = 50 μm. n = 6 for each group (**D**) Quantitative analysis of the Dil-labeled MSC sEVs in lung tissues of mice. The percentages of lung epithelial cells surrounded with Dil-labeled MSC sEVs in CLP + sEVs mice were significantly higher compared with sham + sEVs mice. n = 6 for each group Statistical analysis: Results are presented as mean ± SD, ** P < 0.01, unpaired two-tailed Student’s t-test (**E**) Sections were stained with hematoxylin and eosin and examined histologically. The representative sections are shown at 200 original magnification, and scale bars are 100 μm (**F**) Severity scores of lung injury in the four groups of mice (n = 15). Results are represented as mean ± SD Statistical analysis: one-way ANOVA with a Tukey-Kramer post hoc test. ** *P* < 0.01, compared between the Sham group and each treated group (LPS + PBS, Sham + MSC-sEVs, LPS + MSC-sEVs). ## *P* < 0.01, compared between the LPS + PBS and the treated group (Sham + MSC-sEVs, LPS + MSC-sEVs). && *P* < 0.01, compared between the Sham + MSC-sEVs and the LPS + MSC-sEVs group

The treatment scheme for C57BL/6J mice is shown in Fig. 3A. Analysis of the Kaplan-Meier survival curves revealed that there was a significant difference between LPS-induced ALI mice and sham mice (30% in the LPS group versus 100% in the saline group, Fig. 3B). MSC sEV treatment significantly improved the survival rate of LPS-induced ALI mice (60% in the LPS + sEVs group versus 30% in the LPS group, Fig. 3B), indicating that MSC sEVs could protect mice against LPS-induced death.

Additionally, we also detected the retention of MSC sEVs in lung tissues. Six hours after administration, Dil-labeled MSC sEVs were observed in the perinuclear region of lung epithelial cells (anti-Podoplanin positive cells) (Fig. 3C). The percentages of lung epithelial cells surrounded with Dil-labeled MSC sEVs in CLP + sEVs mice were significantly higher compared with sham + sEVs mice, as shown in Fig. 3D.

Similarly, lungs from mice administered LPS showed alveolar wall thickening, infiltrated inflammatory cells in the alveoli and enlarged interstitial space, while lungs from mice administered MSC sEVs and LPS were devoid of these changes (Fig. 3E). The lung injury scores in mice administered LPS were significantly higher than those in mice administered saline (Fig. 3F). However, MSC sEV treatment significantly reduced the lung injury scores (Fig. 3F).

### MSC sEVs attenuated lung vascular permeability and inflammation in LPS-induced ALI mice

**Fig. 4:**
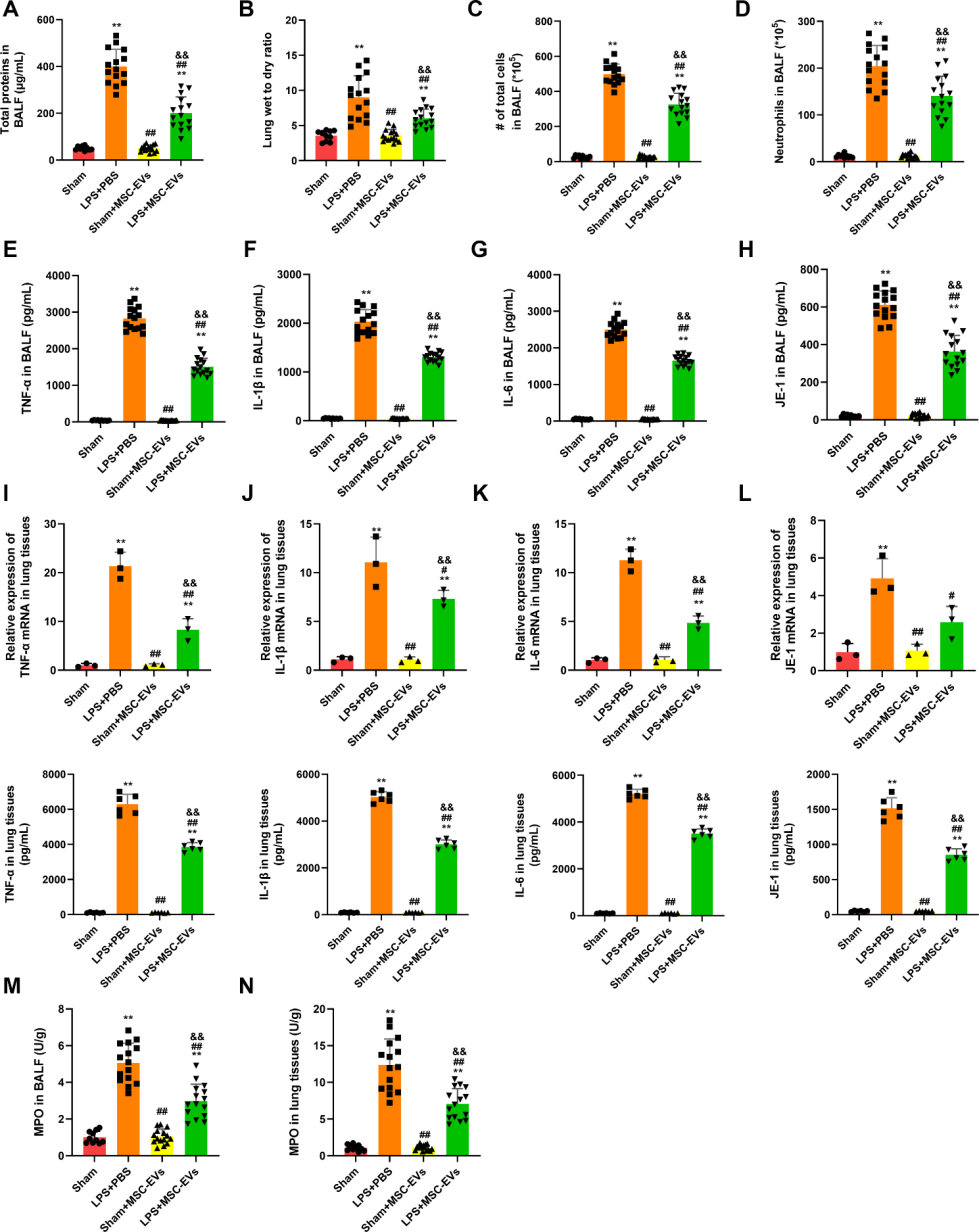
MSC small extracellular vesicles (sEVs) attenuated lung vascular permeability and inflammation in LPS-induced ALI mice (**A-B**) Pulmonary vascular permeability was assessed by measurement of (**A**) total protein in bronchoalveolar fluid (BALF) and (**B**) lung wet/dry ratios (n = 15) (**C-D**) (**C**) Total cells counts and (**D**) neutrophil counts were performed on BALF to evaluate lung inflammation (n = 15) (**E-H**) The levels of proinflammatory cytokines such as (**E**) tumor necrosis factor alpha (TNF-α), (**F**) interleukin-1β (IL-1β), (**G**) interleukin-6 (IL-6), and chemokines (**H**) mouse macrophage chemoattractant protein-1 (JE-1) were measured by mouse enzyme-linked immunosorbent assay (ELISA) in BALF samples (n = 15) (**I-L**) mRNA and protein levels of (**I**) tumor necrosis factor alpha (TNF-α), (**J**) interleukin-1β (IL-1β), (**K**) interleukin-6 (IL-6), and chemokines (**L**) mouse macrophage chemoattractant protein-1 (JE-1) in lung tissues of LPS mice which were treated with or without MSC sEVs (n = 3 for mRNA and n = 6 for protein) (**M-N**) MPO activity in (**M**) BALF and (**N**) lung tissues of saline or ALI mice treated with or without MSC sEVs (n = 15) Statistical analysis: one-way ANOVA with a Tukey-Kramer post hoc test. ** *P* < 0.01, compared between the Sham group and each treated group (LPS + PBS, Sham + MSC-sEVs, LPS + MSC-sEVs). # *P* < 0.05, ## *P* < 0.01, compared between the LPS + PBS and the treated group (Sham + MSC-sEVs, LPS + MSC-sEVs). && *P* < 0.01, compared between the Sham + MSC-sEVs and the LPS + MSC-sEVs group

Lung vascular permeability was assessed by measuring the total protein in BALF and lung wet/dry ratios. LPS intratracheal instillation significantly increased both the total protein in BALF and the lung wet/dry ratios (Fig. 4A-B). Treatment with MSC sEVs markedly reversed the increase in ALI mice challenged with LPS (Fig. 4A-B). Lung inflammation was evaluated by total cell and neutrophil counts in BALF, MPO, and cytokines and chemokines in BALF and pulmonary tissues. Regarding BALF samples, we found that total cell (Fig. 4C), neutrophil (Fig. 4D), proinflammatory cytokine (TNF-a, IL-1β and IL-6) and chemokine JE (mouse MCP-1) (Fig. 4E-H) levels were significantly increased in LPS-induced ALI mice compared with sham mice. We also found that proinflammatory cytokines (TNF-a, IL-1β and IL-6) and the chemokine JE (mouse MCP-1) were significantly augmented in both mRNA and protein levels in pulmonary tissues of LPS-induced mice (Fig. 4I-L). Additionally, we observed that MPO activity was significantly elevated in BALF (Fig. 4M) pulmonary tissues of LPS-induced mice (Fig. 4N). However, the administration of MSC sEVs significantly prevented this LPS-induced increase (Fig. 4A-N). The results suggested that MSC sEVs conferred protection against LPS-induced lung inflammation.

### MiR-223-3p targeted poly [adenosine diphosphate (ADP)–ribose] polymerase–1 (PARP-1)

**Fig. 5:**
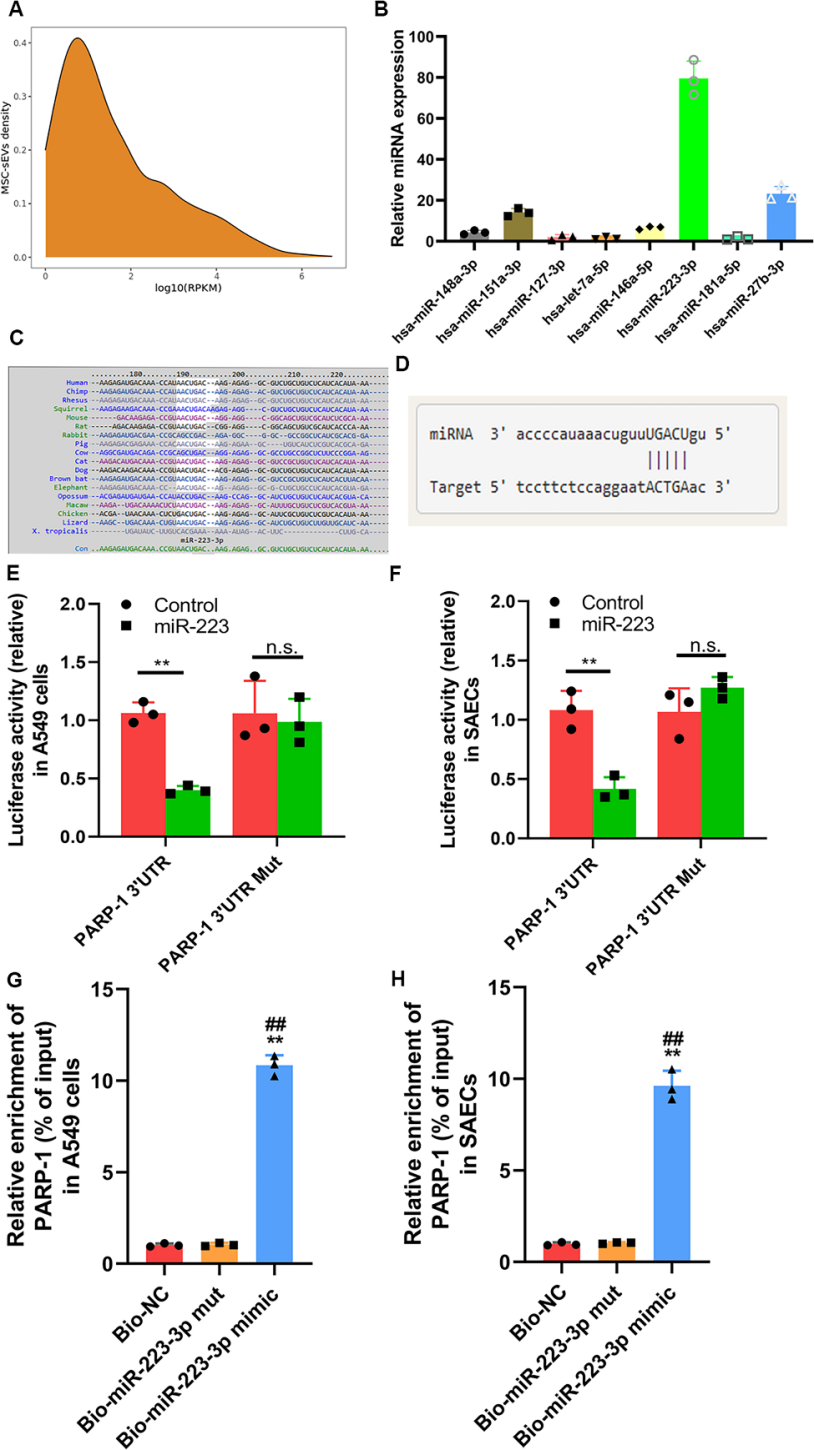
miR-223-3p is abundantly expressed in MSC small extracellular vesicles (sEVs) and could target PARP-1 (**A**) MiRNA expression spectrum in sEVs derived from human umbilical cord Wharton’s jelly-derived MSCs were analyzed by high-throughput sequencing (**B**) High expression of miR-223-3p in human umbilical cord Wharton’s jelly-derived MSCs was measured by qRT-PCR (**C**) miR-223-3p is also an evolutionarily conserved miRNA. (**D**) Bioinformatic analysis predict that the 3′UTRs of PARP-1 are complementary to the miR-223-3p seed region (**E-F**) Luciferase activity of PARP-1-wt and PARP-1-mut was determined upon miR-223-3p mimic transfection in (**E**) A549cells and (**F**) SAECs (n = 3). Statistical analysis: one-way ANOVA with a Tukey-Kramer post hoc test. ** *P* < 0.01, compared between the miR-223-3p mimic transfection group and the control group (**G-H**) The enrichment of miR-223-3p detected by RNA pull-down assay in (**G**) A549 cells and (**H**) SAECs. Statistical analysis: one-way ANOVA with a Tukey-Kramer post hoc test. ** *P* < 0.01, compared with the Bio-NC group. ## *P* < 0.01, compared with the Bio-miR-223-3p mut group

MiRNAs have been reported to contribute to mediating cellular communication. To explore the underlying molecular mechanisms of MSC sEVs in ALI, we performed miRNA sequencing to evaluate the expression profiles of miRNAs encapsulated in MSC (Figure S6, supplementary files 2 and 4) and MSC-sEVs (Fig. 5A, supplementary files 3 and 4). According to the expression levels, the top 8 highly expressed miRNAs were identified in MSC sEVs, including miR-148a-3p, miR-148a-3p, miR-127-3p, let-7a-5p, miR-146a-5p, miR-223-3p, miR-181a-5p, and miR-27b-3p. The qRT-PCR data also confirmed that miR-223-3p was enriched in MSC sEVs (Fig. 5B). Reis et al. [39] and Liu et al. [40]. also showed that miR-223-3p was highly expressed in hUC-MSCs sEVs. Moreover, miR-223-3p was demonstrated to play an important role in inflammation [41]. miR-223-3p is also an evolutionarily conserved miRNA (Fig. 5C), and its expression pattern during anti-inflammation is similar in human and mouse systems. Therefore, we focused on miR-223-3p for further study.

We performed bioinformatics analysis (miRTarBase, ElMMo, miRDB and microT) to predict the potential targets of miR-223-3p (Figure S7). Among these candidates, we selected PARP-1 (Fig. 5D) and NFIA. To validate that both the 3ʹ-UTRs of PARP-1 and NFIA were direct targets of miR-223-3p, we conducted dual-luciferase reporter gene assays. Luciferase activity analysis showed that miR-223-3p dramatically decreased luciferase expression in A549 cells and SAECs cocultured with the 3′‐UTR-WT-PARP-1 vector, but the inhibitory effect was not observed in those cocultured with the 3′‐UTR-MUT-PARP-1 vector (Fig. 5E-F). The similar results were observed in A459 cell when cocultured with the 3′‐UTR-MUT-NFIA vector (Figure S8). Moreover, the RNA pull-down assay showed that both PARP-1 was enriched by biotinylated miR-223-3p, proving their direct interaction (*P* < 0.01, Fig. 5G-H).

### MSC sEVs increased miR-223-3p while decreasing the expression of its target gene PARP-1 in both lung epithelial cells and lung tissues

**Fig. 6:**
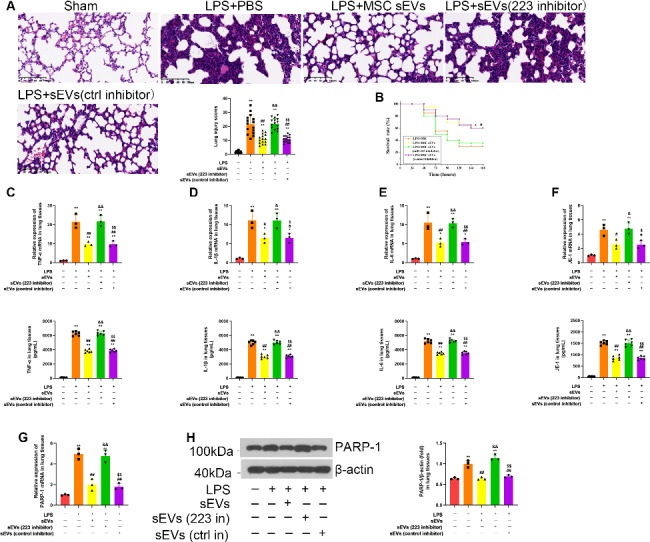
MSC small extracellular vesicles (sEVs) increased miR-223-3p while decreasing the expression of its target gene PARP-1 expression in both lung epithelial cells and lung tissues (**A-C**) The levels of miR-223-3p in LPS stimulated (**A**) A549 cells and (**B**) SAECs after coculturing with or without MSC sEVs. (**C**) The levels of miR-223-sp in lung tissues of LPS mice which were treated with or without MSC sEVs. (n = 3) (**D-F**) The mRNA levels of PARP-1 in LPS stimulated (**D**) A549 cells and (**E**) SAECs after coculturing with or without MSC sEVs. (**F**) The mRNA levels of PARP-1 in lung tissues of LPS mice which were treated with or without MSC sEVs. (n = 3) (**G-I**) The protein levels of PARP-1 in LPS stimulated (**G**) A549 cells and (**H**) SAECs after coculturing with or without MSC sEVs. (**I**) The protein levels of PARP-1 in lung tissues of LPS mice which were treated with or without MSC sEVs. (n = 3). Full-length blots/gels are presented in Supplementary Files 5 Statistical analysis: one-way ANOVA with a Tukey-Kramer post hoc test. ** *P* < 0.01, compared between the Sham group and each treated group (LPS + Saline, Sham + sEVs, LPS + sEVs). # *P* < 0.05, ## *P* < 0.01, compared between the LPS + Saline and the treated group (Sham + sEVs, LPS + sEVs). & *P* < 0.05, && *P* < 0.01, compared between the Sham + sEVs and the LPS + sEVs group

In vitro, in both LPS-induced A549 cells and SAECs, treatment with MSC sEVs significantly elevated the expression of miR-223 compared with those without MSC sEV treatment (Fig. 6A-B). Furthermore, we found that both the mRNA and protein levels of PARP-1 in A549 cells and SAECs cells were significantly reduced upon MSC sEV treatment (Fig. 6D-E and G-H, supplementary file 5). However, the mRNA levels of NFIA in A549 cells was only slightly reduced upon MSC sEV treatment, which was significantly higher than that of PARP-1 (Figure S9). Therefore, we selected PARP-1 for further study.

In vivo, the results showed that in both LPS-induced and sham mice, treatment with MSC sEVs significantly increased miR-223 expression levels compared with untreated mice (Fig. 6C). In contrast, the expression of PARP-1 was markedly suppressed in pulmonary tissues of MSC sEV-treated mice compared with untreated mice (Fig. 6F, I, supplementary file 5). The above results suggest that MSC sEVs may transfer miR-223 to recipient cells and modulate the expression of its target gene PARP-1.

### MSC sEVs suppressed LPS-induced inflammation in lung epithelial cells and lung tissues through the delivery of miR-223-3p

**Fig. 7:**
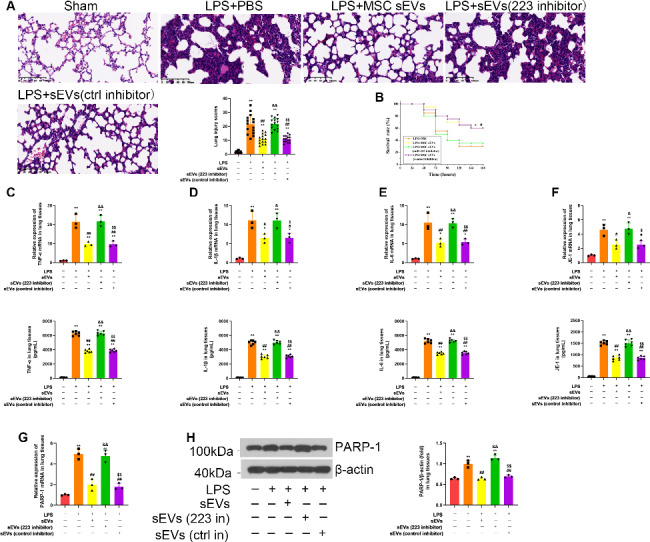
Mesenchymal stem cell small extracellular vesicles (MSC sEVs) treatment suppressed inflammation in lung epithelial cells through the delivery of miR-223-3p Lung epithelial cells were cocultured with MSC sEVs which were additionally treated with a specific inhibitor targeting miR-223-3p (**A-D**) The mRNA and protein expressions of proinflammatory cytokines (**A**) tumor necrosis factor alpha (TNF-α), (**B**) interleukin-1β (IL-1β), (**C**) interleukin-6 (IL-6) and chemokines (**D**) macrophage chemoattractant protein-1 (MCP-1) in A549 cells were determined by qRT-PCR analysis and enzyme-linked immunosorbent assay (ELISA), respectively. n = 3 for mRNA and n = 6 for protein in each group (**E-H**) The mRNA and protein expressions of proinflammatory cytokines (**E**) tumor necrosis factor alpha (TNF-α), (**F**) interleukin-1β (IL-1β), (**G**) interleukin-6 (IL-6) and chemokines (**H**) macrophage chemoattractant protein-1 (MCP-1) in SAECs were determined by qRT-PCR analysis and enzyme-linked immunosorbent assay (ELISA), respectively. n = 3 for mRNA and n = 6 for protein in each group (**I-J**) The mRNA expressions of PARP-1 in (**I**) A549 cells and (**J**) SAECs were determined by qRT-PCR analysis. n = 3 per group (**K-N**) The protein expressions of PARP-1 in (**K, M**) A549 cells and (**L, N**) SAECs were measured by western blot. n = 3 per group. Full-length blots/gels are presented in Supplementary Files 5. Statistical analysis: one-way ANOVA with a Tukey-Kramer *post hoc* test. Results are represented as mean ± SD. * *P* < 0.05, ** *P* < 0.01, compared with the Sham group. # *P* < 0.05, ## *P* < 0.01, compared with the LPS + PBS group. & *P* < 0.05, && *P* < 0.01, compared with the LPS + sEVs group. $ *P* < 0.05, $$ *P* < 0.01, compared with the LPS + sEVs (223 inhibitor) group

To elucidate the effect of MSC sEVs on LPS-induced ALI, we cocultured A549 cells or SAECs with MSC sEVs that were either treated with the miR-223-3p inhibitor or not. In vitro, we found that MSC sEVs dramatically suppressed both the mRNA and protein levels of proinflammatory cytokines (TNF-a, IL-1β and IL-6), the chemokine MCP-1 and the miR-223-3p targeting gene PARP-1 in LPS-challenged A549 cells (Fig. 7A-D, I, K and M, supplementary file 5) or SAECs (Fig. 7E-H, J, L and N, supplementary file 5). However, miR-223 inhibitor-treated MSC sEVs markedly reversed these reductions.

**Fig. 8:**
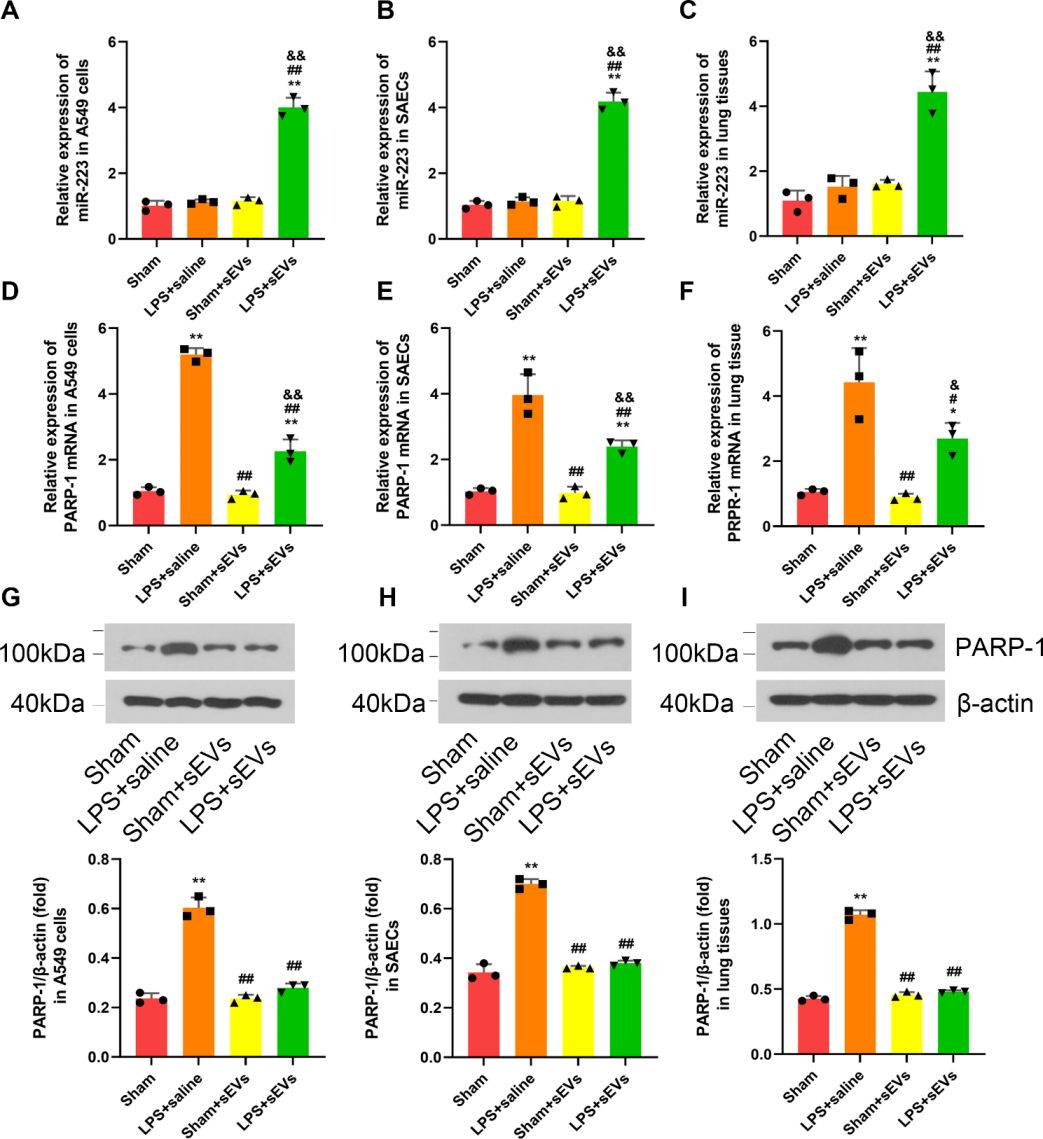
Mesenchymal stem cell small extracellular vesicles (MSC sEVs) treatment suppressed inflammation in lung tissues of LPS-induced ALI mice through the delivery of miR-223-3p LPS-induced ALI mice were treated with Saline, MSC sEVs, or miR-223-3p-depleted MSC sEVs. (**A**) Sections were stained with hematoxylin and eosin and examined histologically. The representative sections are shown at 200 original magnification, and scale bars are 100 μm. Severity scores of lung injury in the four groups of mice (n = 15). Results are represented as mean ± SD. (**B**) Survival rate was monitored for a total of 168 h (7 days) for LPS-induced mice. (*P < 0.05, compared with the LPS + PBS group; #*P* < 0.05, compared with the LPS + sEVs (223-3p inhibitor)). n = 20 per group (**C-F**) The mRNA and protein expressions of proinflammatory cytokines (**C**) tumor necrosis factor alpha (TNF-α), (**D**) interleukin-1β (IL-1β), (**E**) interleukin-6 (IL-6) and chemokines (**F**) mouse macrophage chemoattractant protein-1 (JE-1) in lung tissues of LPS-induced ALI mice. n = 3 for mRNA and n = 6 for protein in each group (**G-H**) (**G**) The mRNA and (**H**) protein expressions of PARP-1 in lung tissues of LPS-induced ALI mice. n = 3 per group. Full-length blots/gels are presented in Supplementary Files 5 Statistical analysis: one-way ANOVA with a Tukey-Kramer *post hoc* test. Results are represented as mean ± SD. * *P* < 0.05, ** *P* < 0.01, compared with the Sham group. # *P* < 0.05, ## *P* < 0.01, compared with the LPS + PBS group. & *P* < 0.05, && *P* < 0.01, compared with the LPS + sEVs group. $ *P* < 0.05, $$ *P* < 0.01, compared with the LPS + sEVs (223-3p inhibitor) group

In vivo, similar trends were observed for the mRNA and protein levels of proinflammatory cytokines (TNF-a, IL-1β and IL-6), the chemokine MCP-1 and the miR-223-3p targeting gene PARP-1 in the lung tissues of LPS-induced ALI mice (*P* < 0.01, Fig. 8C–H, supplementary file 5). Moreover, miR-223-3p inhibitor-treated MSC sEVs also significantly reversed the MSC sEV-induced beneficial effect on the survival rate and lung injury scores of ALI mice (Fig. 8A-B).

These data indicate that MSC sEVs may suppress LPS-induced inflammation by targeting PARP-1 through miR-223-3p.

### Inhibition of PARP-1 induces MSC-sEV-like positive effects on lung epithelial cells

**Fig. 9:**
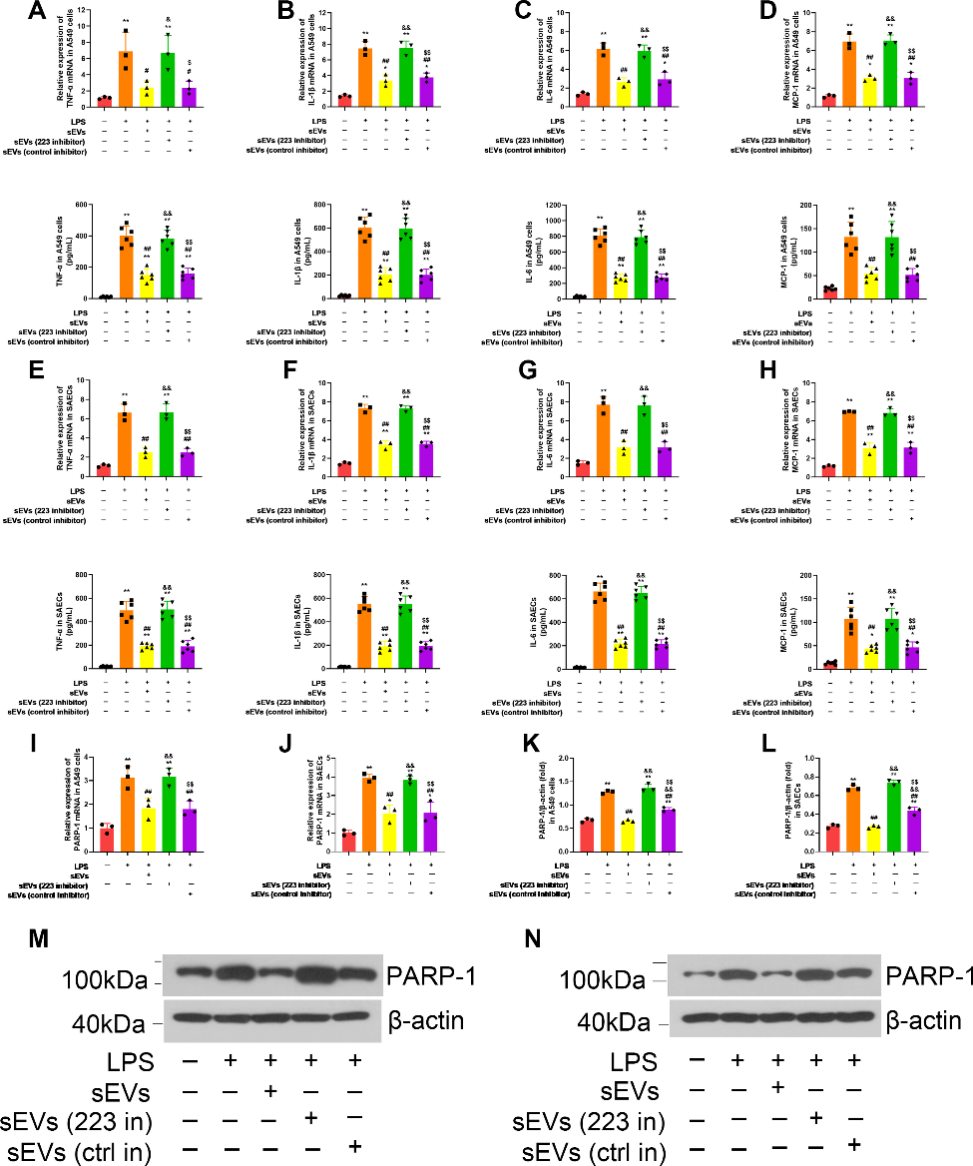
Inhibition of PARP-1 induce mesenchymal stem cell small extracellular vesicles (MSC sEVs) like positive effects on lung epithelial cells Lung epithelial cells were cocultured with MSC sEVs or siPARP-1. (**A-B**) The inhibitory efficiency of the siRNAs targeting PARP-1 in (**A**) A549 cells and (**B**) SAECs was verified by qRT-PCR. n = 3 per group (**C-F**) The mRNA and protein expressions of proinflammatory cytokines (**C**) tumor necrosis factor alpha (TNF-α), (**D**) interleukin-1β (IL-1β), (**E**) interleukin-6 (IL-6) and chemokines (**F**) macrophage chemoattractant protein-1 (MCP-1) in A549 cells (**G-J**) The mRNA and protein expressions of proinflammatory cytokines (**G**) tumor necrosis factor alpha (TNF-α), (**H**) interleukin-1β (IL-1β), (**I**) interleukin-6 (IL-6) and chemokines (**J**) macrophage chemoattractant protein-1 (MCP-1) in SAECs. n = 3 for mRNA and n = 6 for protein in each group Statistical analysis: one-way ANOVA with a Tukey-Kramer *post hoc* test For (**A**) and (**B**), ** *P* < 0.01, compared with the con siRNA group. For (C-J), * *P* < 0.05, ** *P* < 0.01, compared with the control group. # *P* < 0.05, ## *P* < 0.01, compared with the LPS + PBS group. & *P* < 0.05, && *P* < 0.01, compared with the LPS + sEVs group. $ *P* < 0.05, $$ *P* < 0.01, compared with the LPS + siPRAP-1 group. Results are represented as mean ± SD.

To further explore the mechanism of the beneficial effect of MSC sEVs on LPS-induced ALI, we used siRNA to knock down the expression of the miR-223-3p target gene PARP-1. As shown in Fig. 9A-B, qRT-PCR analysis showed that the most effective siRNA was siPARP-1 #1. Therefore, siPARP-1 #1 was selected for the following experiments. We found that inhibition of PARP-1 markedly reduced the mRNA and protein levels of proinflammatory cytokines (TNF-a, IL-1β and IL-6) and the chemokine MCP-1 in LPS-induced A549 cells (Fig. 9C-F) or SAECs (Fig. 9G-J), which was similar to the effect of treatment with MSC sEVs. These results further confirmed that the beneficial effects of MSC sEVs on the inflammation of lung epithelial cells may be related to miR-223-3p by targeting PARP-1.

## Discussion

ALI/ARDS is a life-threatening disease with high morbidity and mortality [3, 4]. The pathophysiology of ALI/ARDS is diffuse alveolar and endothelial damage, resulting in acute overwhelming inflammatory reactions in the lungs [42]. In this study, we found that sEVs derived from MSCs successfully improved the survival rate, decreased pulmonary capillary membrane permeability, and attenuated inflammation of lung epithelial cells in LPS-induced ALI mice. We observed that the beneficial effect of MSC sEVs on ALI was partly eliminated when miR-223 expression was inhibited in MSCs. Notably, we found that MSC sEV treatment markedly upregulated miR-223-3p expression while downregulating the expression of the miR-223-3p targeting gene PARP-1 and subsequently reducing LPS-induced inflammation in lung epithelial cells.

Recently, numerous studies have demonstrated that MSCs have therapeutic potential in different preclinical lung inflammation ALI models (LPS, influenza, Pseudomonas aeruginosa and Escherichia coli) [43–49]. Additionally, MSCs have been reported to play beneficial roles in randomized phase I/II clinical trials of ARDS [50–52]. Our in vitro data also showed that MSCs could suppress LPS-induced inflammation in lung epithelial cells. Recently, miRNAs have been found to play crucial roles in the amelioration of various inflammation-induced diseases, including ALI [53]. To explore mechanisms underlying the beneficial effects, miRNA-seq were performed on MSCs (Figure S3, supplementary files 2). Several miRNAs were found to be enriched in MSCs, such as miR-126-3p, miR-181a-5p, miR-146-5p, miR-455-3p, miR-133a and so on. These microRNA were demonstrated to show protective role in repairing lung injury as well as other organ injury. For example, miR-126-3p was reported to attenuate LPS-induced ALI [54] and be an effective therapeutic strategy for premature ovarian failure [55]. MiR-181a-5p was showed to attenuate sepsis-induced acute kidney injury [56] and also severe acute pancreatitis [57]. MiR-146-5p was demonstrated to inhibit pro-inflammatory pathways in sepsis [58] and accelerate diabetic wound healing [59]. MiR-455-3p was revealed to attenuate LPS-induced injury in bronchial epithelial cells [60] and prevent cardiac ischemia-reperfusion injury [61]. MiR-133a was also reported to have a protective effect on sepsis-induced kidney injury [62].

However, there is an increasing concern for MSC therapy because of the high risk of carcinogenesis, limited efficacy of engraftment and differentiation, short-term survival in vivo, and immune rejection [13, 63]. Therefore, increasing attention has been focused on MSC sEVs as a cell-free treatment regimen [64]. Recent research has demonstrated that MSC sEVs can play almost the same positive anti-inflammatory roles as MSCs themselves, which indicates that MSC sEVs may be an ideal candidate for ARDS therapy [65, 66]. Previous reports showed that MSC-derived or endothelial progenitor cell-derived sEVs inhibited the inflammation of macrophage cells in in vitro and in vivo models of ARDS [66–68]. Here, we provide evidence showing that MSC sEVs are also able to suppress the inflammation of lung epithelial cells in LPS-induced ALI, which further confirms the anti-inflammatory role of MSC sEVs.

Although MSC sEVs have been demonstrated to attenuate inflammation in ALI, the mechanisms underlying the beneficial effect of MSC sEVs have not been completely explored. Recent findings have shown that MSC EVs are involved in ALI therapy by transferring mRNA to injured tissue. For example, Zhu et al. and Tang et al. reported that MSC sEVs attenuated inflammation and decreased pulmonary capillary permeability in LPS-induced ALI mice partly by transferring keratinocyte growth factor (KGF) mRNA and angiopoietin-1 (Ang-1) mRNA to injured lung tissues [36, 69]. Remarkably, growing evidence shows that miRNAs are important MSC sEV contents and can regulate the gene expression of recipient cells [70, 71]. MSC sEV-derived miR21-5p [72] or miR-27a-3p [73] was demonstrated to be delivered into alveolar macrophages and subsequently inhibited proinflammatory cytokines or decreased NF-κB expression, respectively. Similarly, MSC sEVs were reported to transfer miR145 into macrophages and reduce the *E. coli* bacterial load in a bacterial pneumonia mouse model [74]. In line with the findings of those studies, we showed that miR-223-3p was significantly increased in LPS-induced lung epithelial cells after treatment with MSC sEVs. The results indicated that miR-223-3p may be transferred from MSC sEVs into lung epithelial cells, which may partially explain the mechanisms of MSC sEVs in ALI therapy.

MiRNAs play crucial roles in multiple biological processes, including cell proliferation, differentiation, development, and apoptosis [75, 76]. Numerous studies have shown that miR-223-3p is involved in the regulation of the inflammatory response and as well as other disease models [29, 77, 78]. It was reported that miR-223-3p contributed to inhibiting NLRP3 protein accumulation and IL-1β production in THP-1 cells [78]. Importantly, Wang et al. showed that MSC sEVs might deliver miR-223 into cardiomyocytes and inhibit inflammation during sepsis in vitro and in vivo [29]. Accumulating evidences have indicated that miR-223-3p could play an important role in diabetic retinopathy [79], non-small cell lung cancer [80], breast cancer [81], etc. In this study, we found that MSC sEV miR-223-3p was involved in the inhibition of the inflammatory response in lung epithelial cells upon LPS challenge. To further explore the underlying mechanisms of MSC sEV miR-223-3p, bioinformatic tools were applied to identify the potential target gene of miR-223-3p. We observed that PARP-1 was a potential target gene of miR-223-3p in the regulation of inflammation in lung epithelium cells. The luciferase reporter assay analysis results confirmed that PARP-1 was a target gene of miR-223-3p. Moreover, we found that both the mRNA and protein levels of PARP-1 were significantly increased in lung epithelial cells in vitro and in vivo after treatment of MSC sEVs with a miR-223-3p inhibitor. This result further confirmed that PARP-1 was the target downstream gene of miR-223-3p. PARP-1 is a well-characterized member of the PARP family, which is comprised of 17 members and single PARP proteins and is involved in key cellular processes such as DNA repair and cell death [82]. Additionally, PARP-1 has been demonstrated to contribute to inflammation and tissue injury [41, 83]. Neudecker et al. reported that neutrophils could transfer sEV miR-223 into lung epithelial cells and subsequently attenuate lung injury by targeting PARP-1 [41]. In agreement with the observation in the above report, we found that MSC sEVs might deliver miR-223-3p to LPS-induced lung epithelium cells and inhibit their inflammation by targeting PARP-1. Importantly, we also observed that knockout of PARP-1 could achieve MSC-sEV-like positive effects in LPS-induced lung epithelium cells. These findings suggest that miR-223-3p transferred by MSC sEVs might provide an anti-inflammatory benefit in lung epithelium cells by targeting PARP-1.

Several limitations of this study should be acknowledged. First, the intratracheal instillation of an LPS mouse model could not fully mimic the pathophysiological process seen in patients with ARDS. Maybe future studies are required to assess the effect of MSC sEV miR-223-3p in other ALI models to validate its clinical significance. Second, how MSC sEV miRNAs are taken up by lung epithelial cells and whether there are other ways to transport miR-223-3p are still unknown. Third, numerous bioactive molecules were demonstrated to be contained in MSC sEVs, and we only focused on miR-223-3p in this study. Therefore, we cannot rule out the possibility that other bioactive molecules within MSC sEVs also account for some of the beneficial effects on inflammation of lung epithelial cells in ALI, which needs further investigation. Fourth, miRNAs usually regulate multiple target genes, and we only focused on one of the miR-223-3p target genes, PARP-1. Therefore, we cannot rule out the possibility that other targeting genes also play an important role in regulating the inflammatory response in lung epithelial cells in ALI.

## Conclusions

**Fig. 10:**
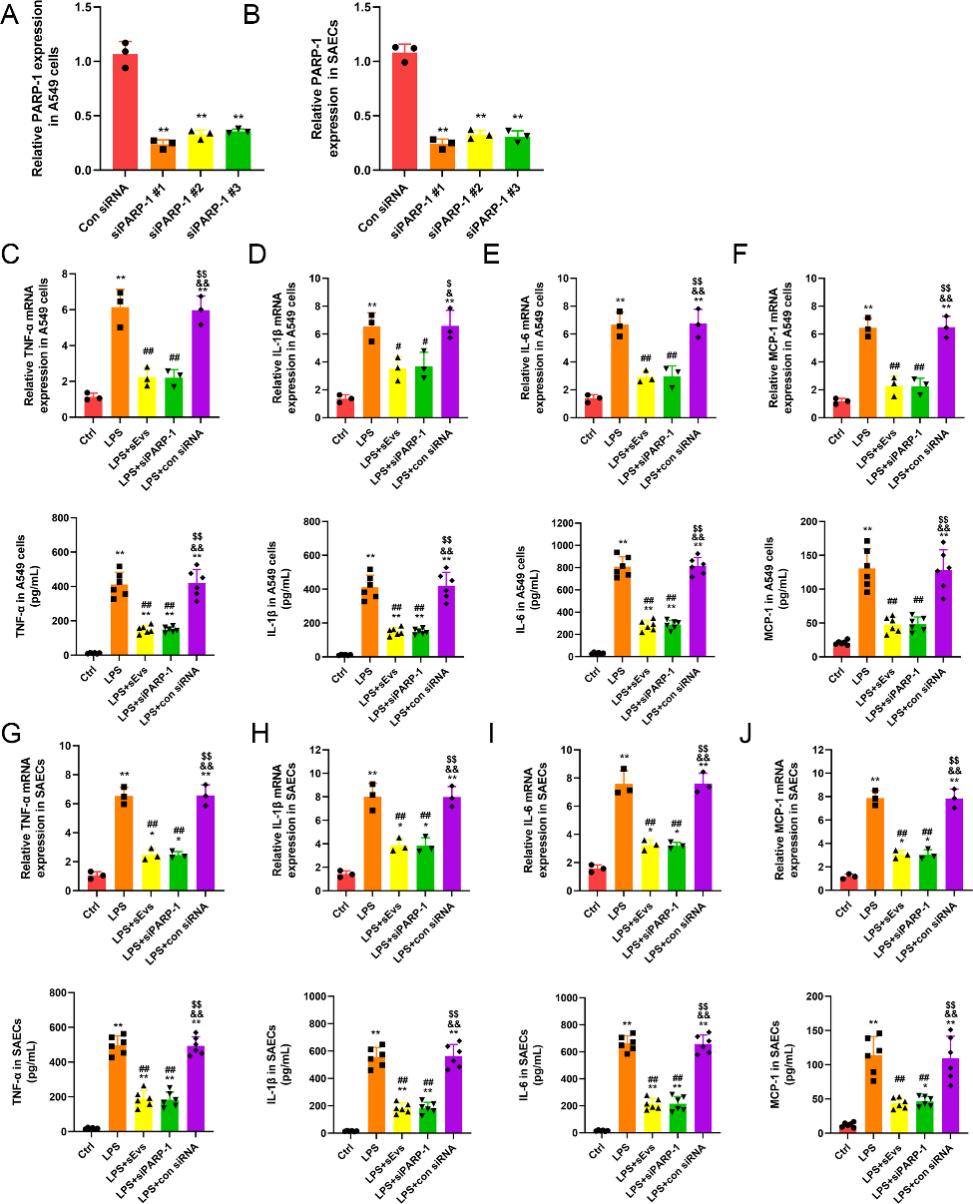
Scheme of the mechanisms involved in the protective effect of MSC sEVs in LPS-induced ALI MSC derived sEVs inhibited the inflammation of LPS-induced ALI may related with miR-223-3p. Moreover, MSC sEV-derived miR-223-3p is likely to suppress inflammation by targeting PARP-1. (This figure was made by ourselves)

In conclusion, the present study shows that MSC sEVs effectively protect the lung from an overwhelming inflammatory response and increase the survival rate in ALI. Our data also suggest that MSC sEVs suppress the inflammation of LPS-induced lung epithelium cells by targeting functional miR-223-3p to lung epithelium cells and then downregulating PARP-1 (Fig. 10). MiR-223-3p in MSC sEVs may represent a promising target for ALI therapy.

### Electronic supplementary material

Below is the link to the electronic supplementary material.

Supplementary Material 1

Supplementary Material 3

Supplementary Material 4

Supplementary Material 5

Supplementary Material 6

**Table S2**: Quantities and quality of small extracellular vesicles from cultures of MSCs

Supplementary Material 8

Supplementary Material 9

Supplementary Material 10

Supplementary Material 11

1. Figure 6G-PARP-1. 2. Figure 6H-PARP-1. 3. Figure 6I-PARP-1. 4. Figure 7M-PARP-1. 5. Figure 7N-PARP-1. 6. Figure 8H-PARP-1. 7. Figure S1B-CD81. 8. Figure S1B-TSG101. 9. Figure S1B- Calnexin

Supplementary Material 13

Supplementary Material 14

Supplementary Material 15

## Data Availability

The raw RNA sequence data reported in this paper have been deposited in the Genome Sequence Archive (Genomics, Proteomics & Bioinformatics 2021) in National Genomics Data Center (Nucleic Acids Res 2022), China National Center for Bioinformation / Beijing Institute of Genomics, Chinese Academy of Sciences (GSA-Human: HRA003253) that are publicly accessible at https://ngdc.cncb.ac.cn/gsa-human [84, 85]. The datasets generated and/or analyzed during the current study are available from the corresponding author on reasonable request.

## Abbreviations

ALI: Acute lung injury
AECs: Alveolar epithelial cells
Ang-1: Angiopoietin-1
ANOVA: Analysis of variance
BAL: Bronchoalveolar lavage
BALF: BAL fluid
DMEM: Dulbecco’s modified Eagle’s medium
ECL: Enhanced chemiluminescence
ELISAs: Enzyme-linked immunosorbent assays
EVs: Extracellular vesicles
FBS: Fetal bovine serum
GAPDH: Glyceraldehyde 3-phosphate dehydrogenase
GvHD: Graft-versus-host disease
HE: Hematoxylin and eosin
hUC-MSCs: Human umbilical cord Wharton’s jelly-derived MSCs
IL-1β: Interleukin-1β
IL-6: Interleukin-6
KGF: Keratinocyte growth factor
LPS: Lipopolysaccharide
MCP-1: Macrophage chemoattractant protein-1
microRNAs: miRNAs
MPO: Myeloperoxidase
MSC sEVs: sEVs derived from mesenchymal stromal cells
MSCs: Mesenchymal stromal cells
PARP-1: Poly (adenosine diphosphate-ribose) polymerase-1
PBS: Phosphate-buffered saline
qRT-PCR: Quantitative real-time polymerase chain reaction
SAECs: Small airway epithelial cells
SD: Standard deviation
sEVs: Small extracellular vesicles
siRNAs: Small interfering RNAs
TEM: Transmission electron microscopy
TNF-α: Tumor necrosis factor alpha
TRPS: Tunable resistive pulse sensing
UCs: Umbilical cords
