# Supplementary figures and images for: Human umbilical cord mesenchymal stromal cell small extracellular vesicle transfer of microRNA-223-3p to lung epithelial cells attenuates inflammation in acute lung injury in mice
Source: J Nanobiotechnology. 2023 Aug 25;21:295. doi: 10.1186/s12951-023-02038-3 (PMC10464265; doi:10.1186/s12951-023-02038-3)

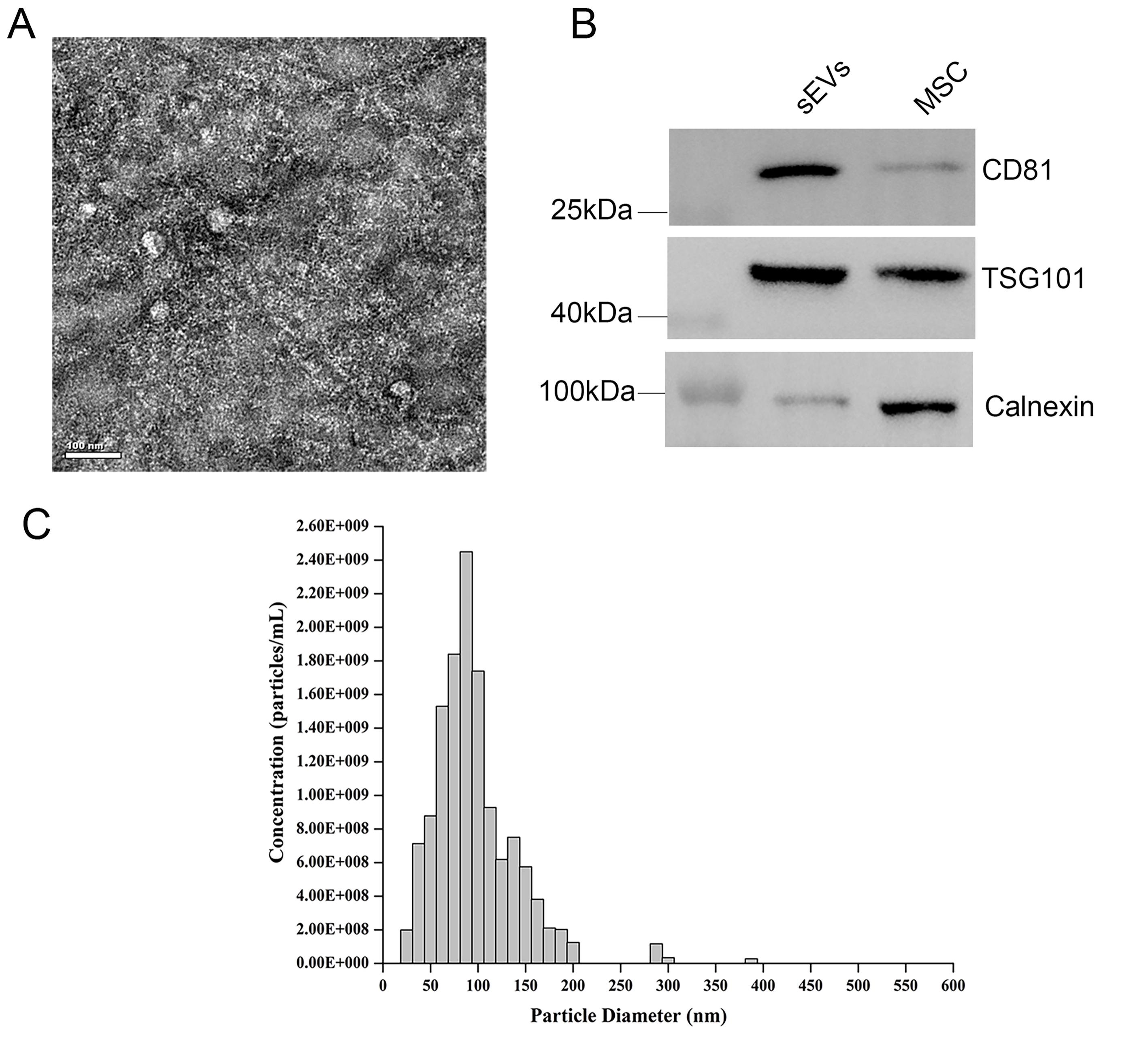

Supplement: Supplementary file 1 — Supplementary Material 1 [file 12951_2023_2038_MOESM1_ESM.png]

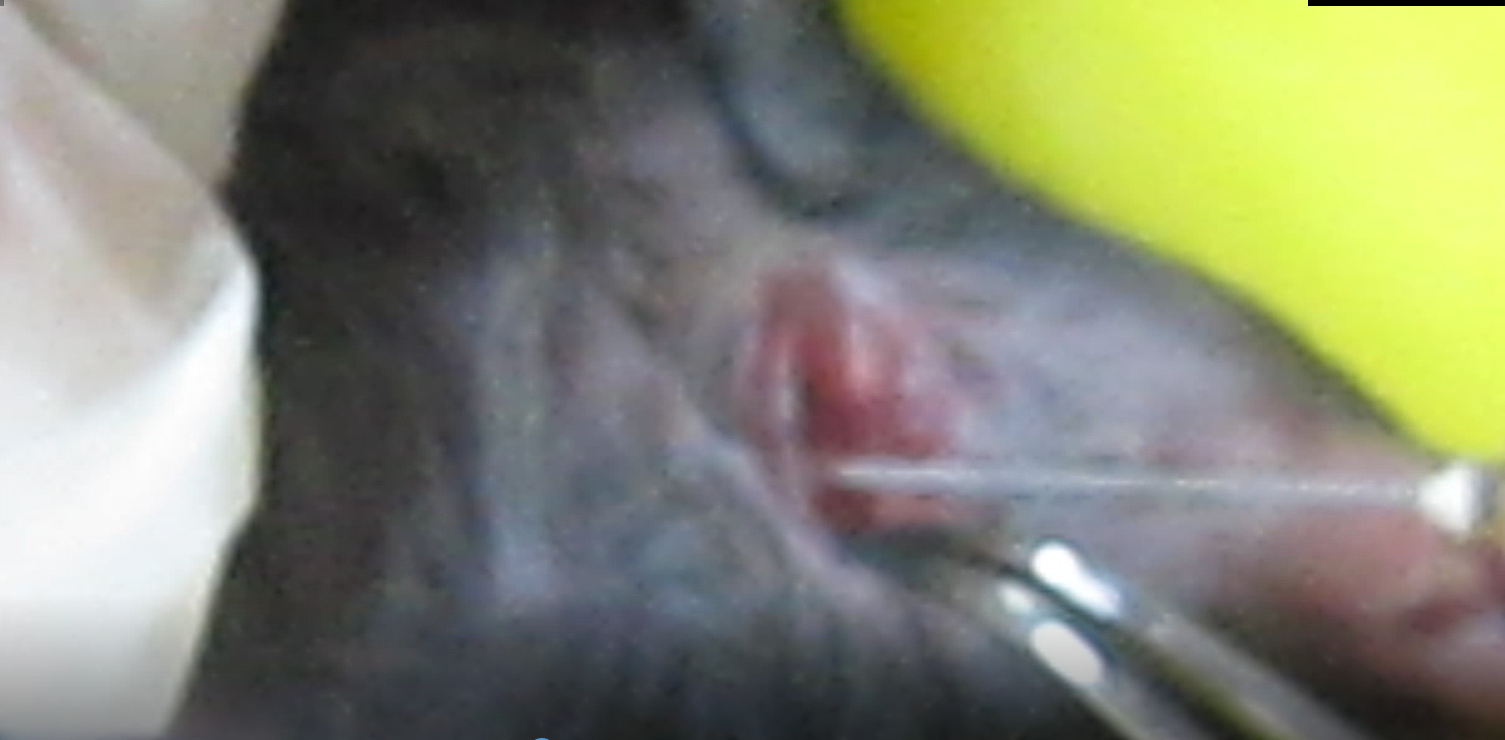

Supplement: Supplementary file 2 — Supplementary Material 3 [file 12951_2023_2038_MOESM3_ESM.png]

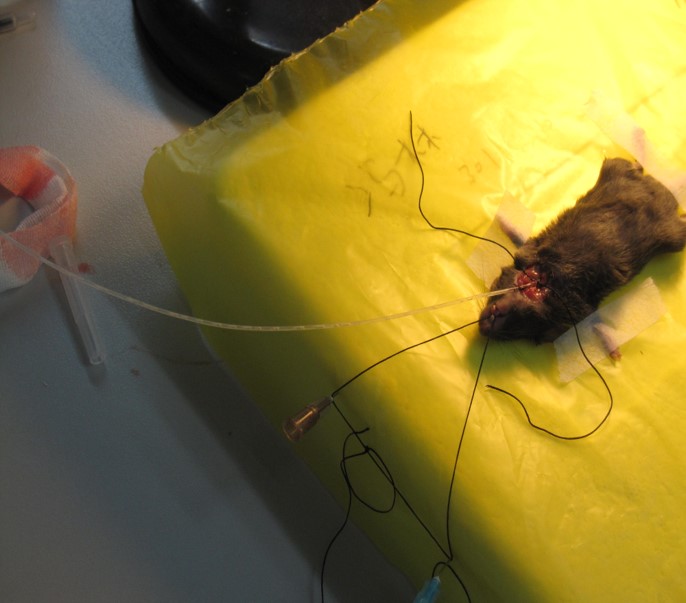

Supplement: Supplementary file 3 — Supplementary Material 4 [file 12951_2023_2038_MOESM4_ESM.jpg]

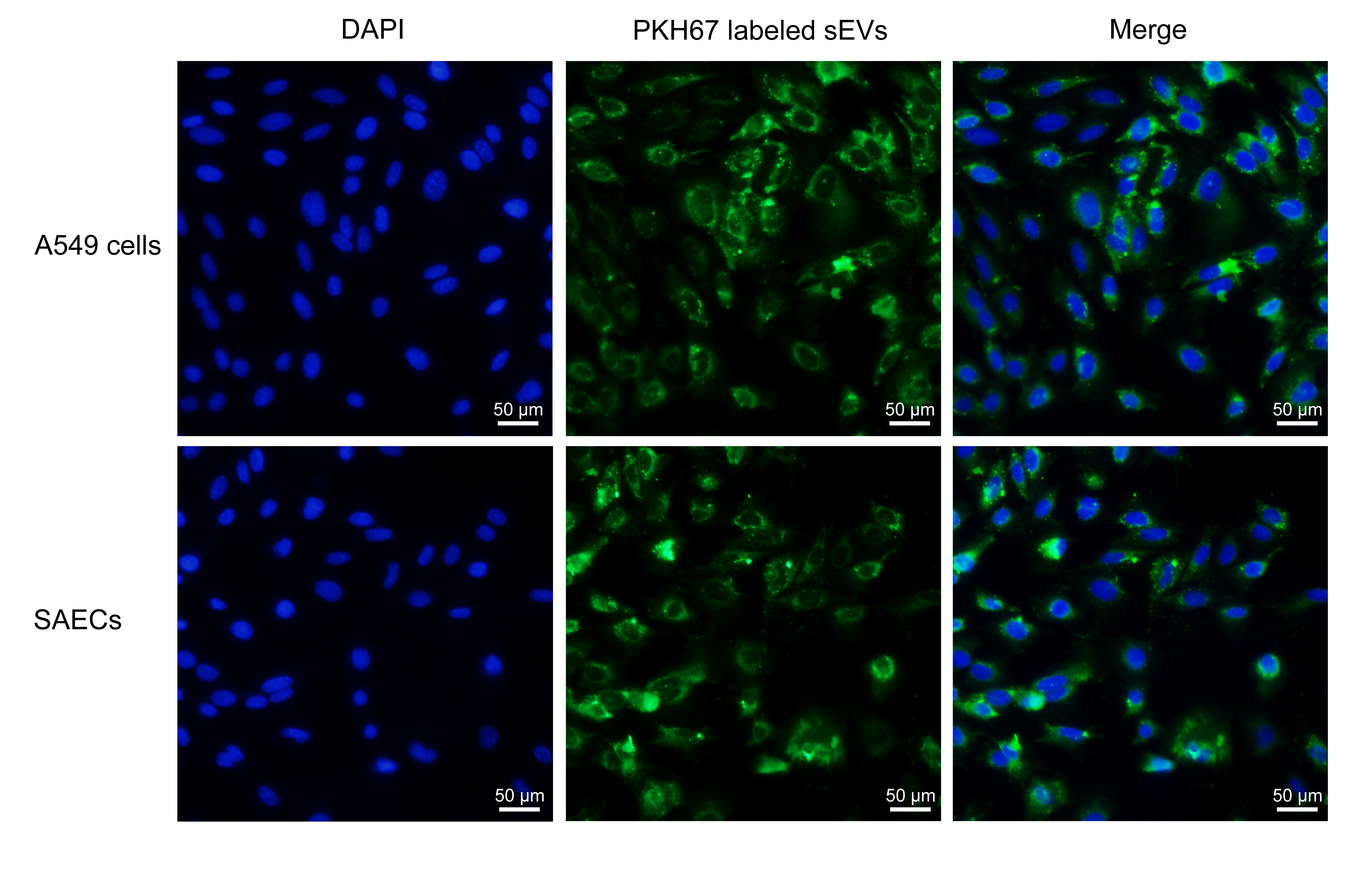

Supplement: Supplementary file 4 — Supplementary Material 5 [file 12951_2023_2038_MOESM5_ESM.png]

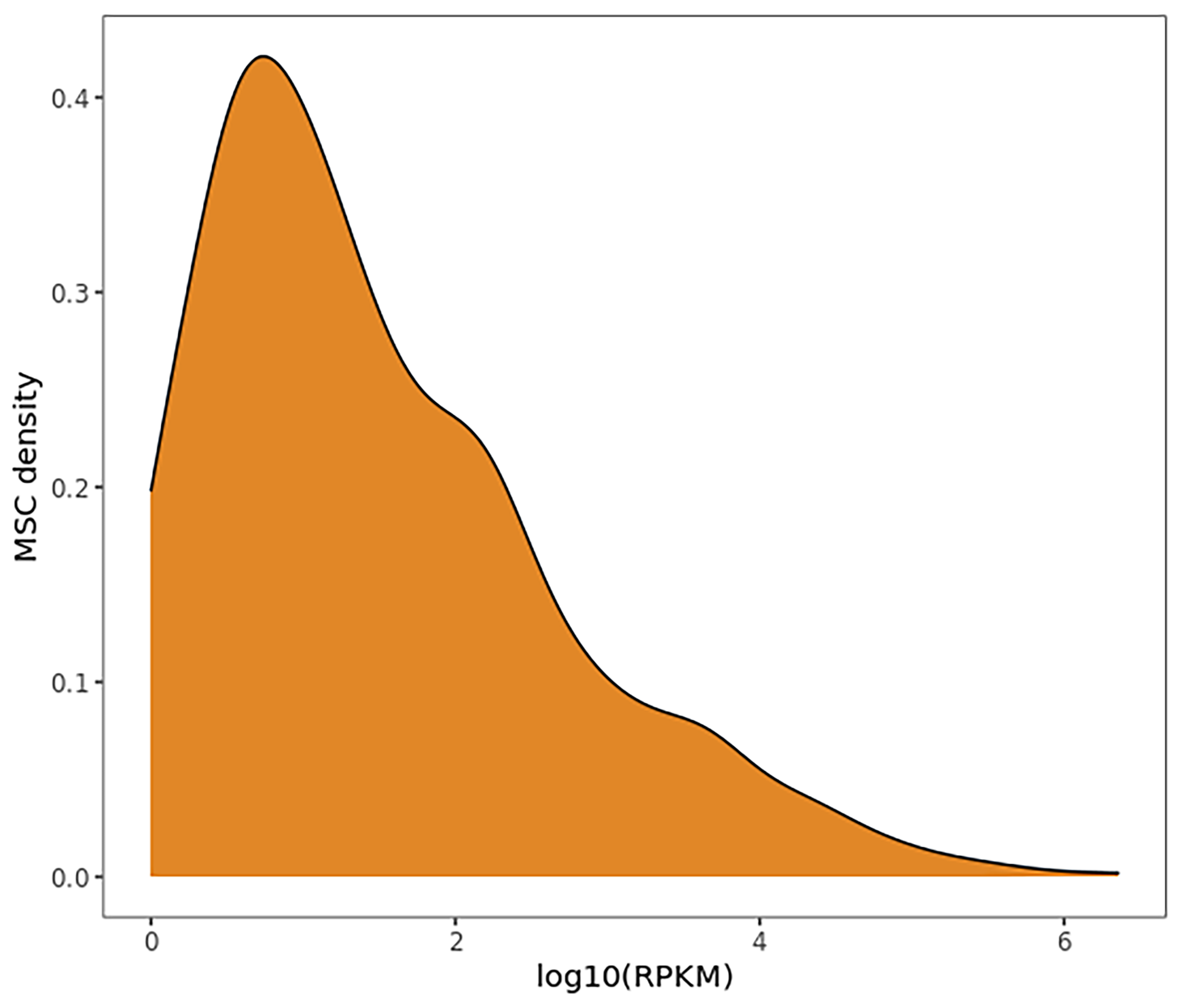

Supplement: Supplementary file 5 — Supplementary Material 6 [file 12951_2023_2038_MOESM6_ESM.png]

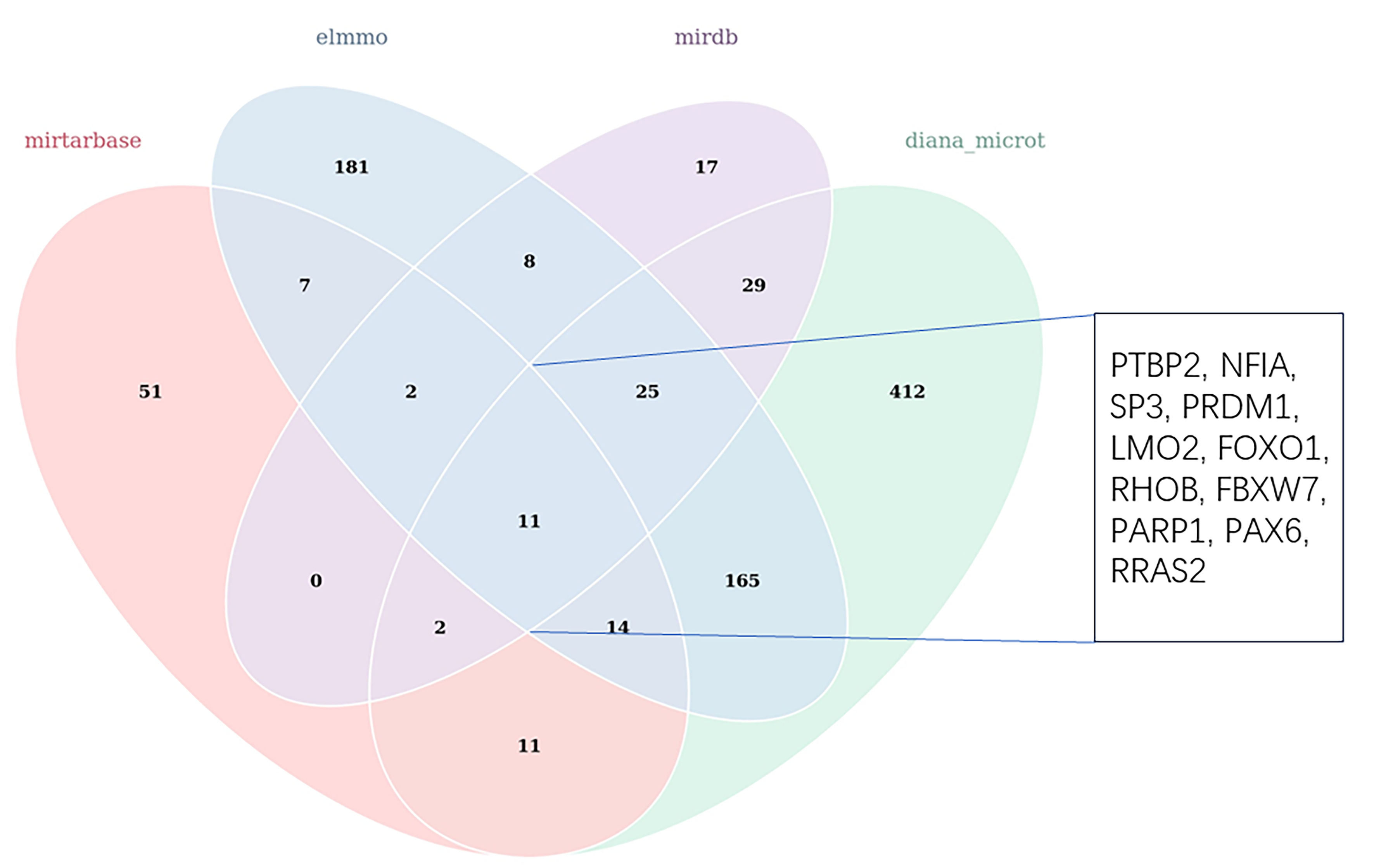

Supplement: Supplementary file 12 — Supplementary Material 13 [file 12951_2023_2038_MOESM13_ESM.png]

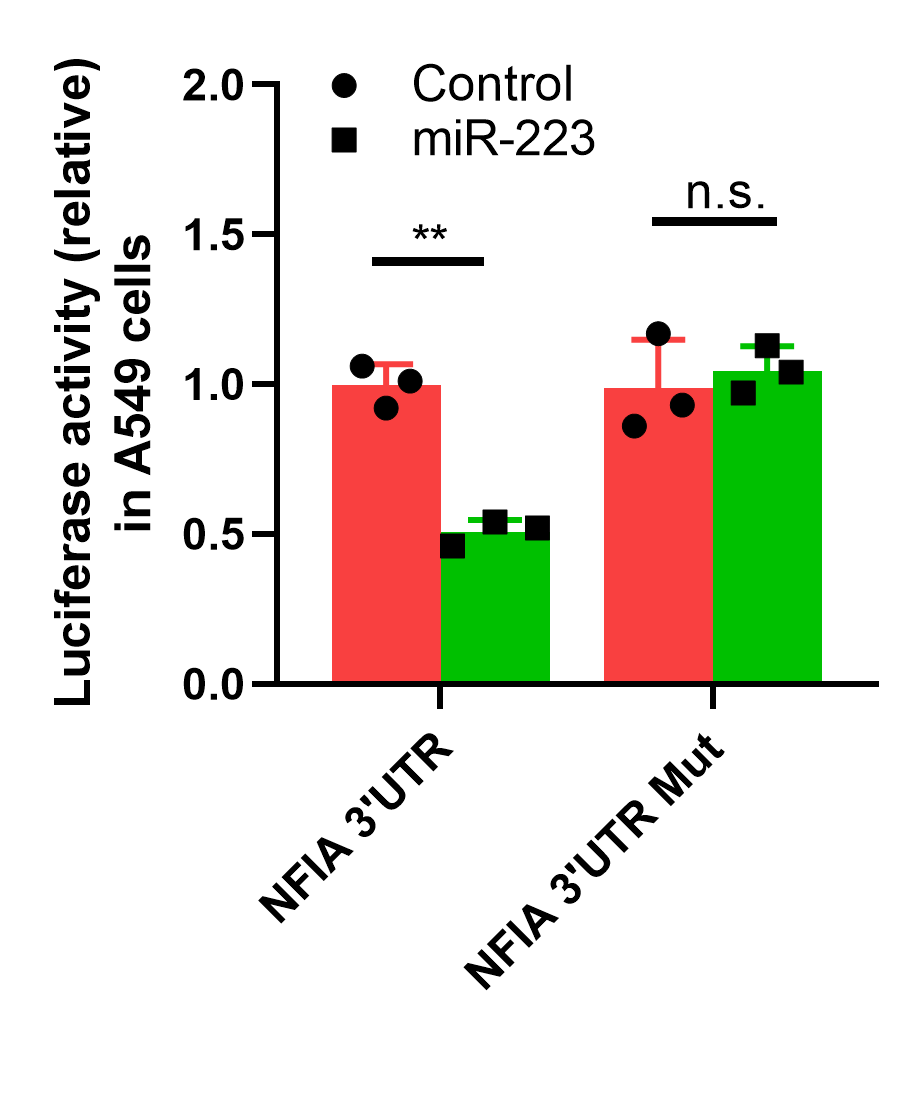

Supplement: Supplementary file 13 — Supplementary Material 14 [file 12951_2023_2038_MOESM14_ESM.png]

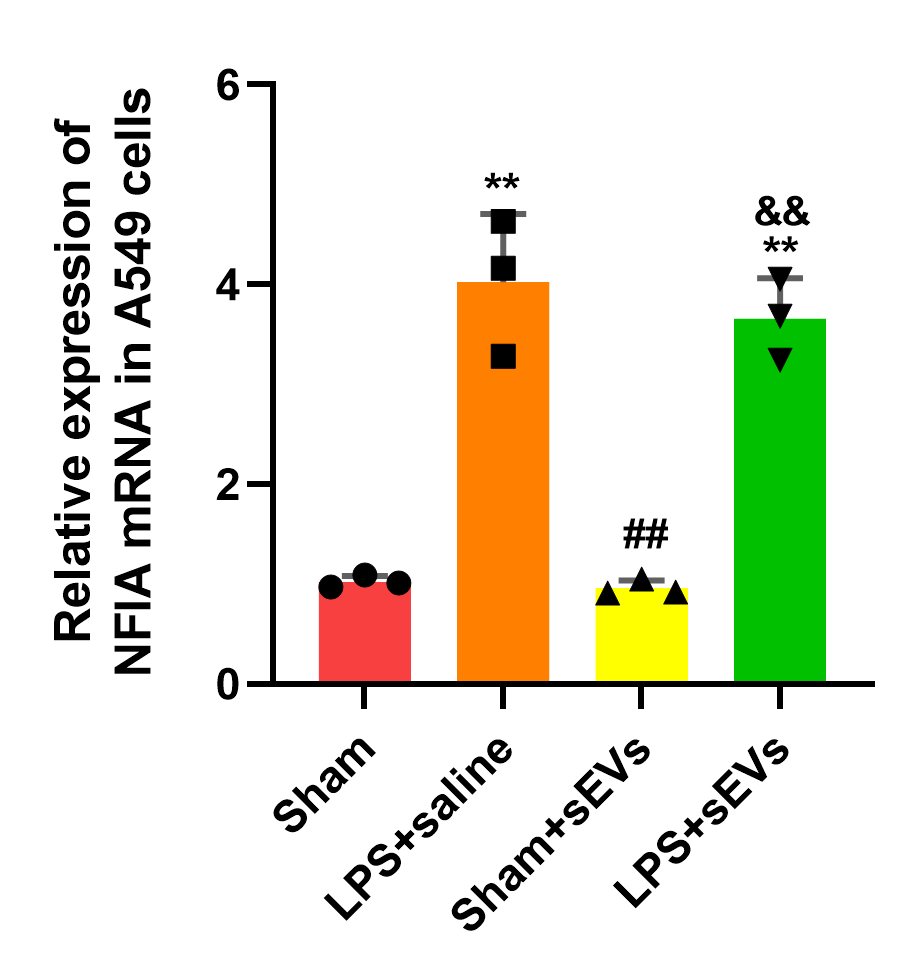

Supplement: Supplementary file 14 — Supplementary Material 15 [file 12951_2023_2038_MOESM15_ESM.png]
