# Supplementary material for: Human umbilical cord mesenchymal stromal cell small extracellular vesicle transfer of microRNA-223-3p to lung epithelial cells attenuates inflammation in acute lung injury in mice
Source: J Nanobiotechnology. 2023 Aug 25;21:295. doi: 10.1186/s12951-023-02038-3 (PMC10464265; doi:10.1186/s12951-023-02038-3)
Supplement: Supplementary file 6 — Table S2: Quantities and quality of small extracellular vesicles from cultures of MSCs [file 12951_2023_2038_MOESM7_ESM.docx]

**Table**

Table S2 Quantities and quality of small extracellular vesicles from cultures of MSCs

|  | No. of MSCs  (×10^6^) | Total number of sEVs  (×10^6^/30μL) | Total sEV  protein  (μg/30μL EV) |
| --- | --- | --- | --- |
| MSCs | 3.00 | 1.89±0.38 | 5.12±0.47 |
