# Supplementary material for: Human umbilical cord mesenchymal stromal cell small extracellular vesicle transfer of microRNA-223-3p to lung epithelial cells attenuates inflammation in acute lung injury in mice
Source: J Nanobiotechnology. 2023 Aug 25;21:295. doi: 10.1186/s12951-023-02038-3 (PMC10464265; doi:10.1186/s12951-023-02038-3)
Supplement: Supplementary file 7 — Supplementary Material 8 [file 12951_2023_2038_MOESM8_ESM.docx]

**Materials and methods**

To maximize the objectivity of the presented analyses, we preregistered this study with its hypotheses, its planned methods, and its complete plan of data analysis before the start of data collection. The details can be found in the Institutional Animal Care and Ethics Committee of Xiangya Hospital. We closely adhered to our plan.

The mice were included in the study if they were successfully intratracheally administered with LPS or saline. The mice were excluded if the mice died prematurely, preventing the collection of plasma and histological data.

The sample size was determined based on the related references (PMID: 20558630) and as well as the survival rate. Power calculations were conducted by the G*Power software (latest ver. 3.1.9.7; Heinrich-Heine-Universität Düsseldorf, Düsseldorf, Germany). Almost all the power was more than 80% in our manuscript.

To avoid bias, mice were divided into three or four groups randomly. The method of randomization was described as follows. Firstly, 40 mice were numbered by the weight. Ear punching was used for identification of each mouse. Five mice which have similar weight were put into one cage. So, there were 8 cages. Random numbers were generated using the standard = RAND () function in Microsoft Excel. The list of copy random number was in ascending order. Then the group A, B, C, D represent the group of Sham, LPS+PBS, Sham+MSC-EVs, LPS+MSC-EVs respectively. Each group had 10 mice. According to the similar randomization method, the 10 mice in each group were divided into two subgroups. The study was repeated three times. The method of randomization for the experiment of mechanism research and survival rate were similar to that used in the above experiments.

For each mouse, five different investigators were involved as follows: a first investigator (SM) administered the treatment based on the randomization table. This investigator was the only person aware of the treatment group allocation. A second investigator (ZGZ) was responsible for the anesthetic procedure, whereas a third investigator (RCN, JC) performed the surgical procedure. Finally, the fourth and fifth investigator (BHL, YQL) (also unaware of treatment) assessed the lung injury scores.
