# Supplementary material for: Human umbilical cord mesenchymal stromal cell small extracellular vesicle transfer of microRNA-223-3p to lung epithelial cells attenuates inflammation in acute lung injury in mice
Source: J Nanobiotechnology. 2023 Aug 25;21:295. doi: 10.1186/s12951-023-02038-3 (PMC10464265; doi:10.1186/s12951-023-02038-3)
Supplement: Supplementary file 11 — 1. Figure 6G-PARP-1. 2. Figure 6H-PARP-1. 3. Figure 6I-PARP-1. 4. Figure 7M-PARP-1. 5. Figure 7N-PARP-1. 6. Figure 8H-PARP-1. 7. Figure S1B-CD81. 8. Figure S1B-TSG101. 9. Figure S1B- Calnexin [file 12951_2023_2038_MOESM12_ESM.docx]

**1. Figure 6G-PARP-1**


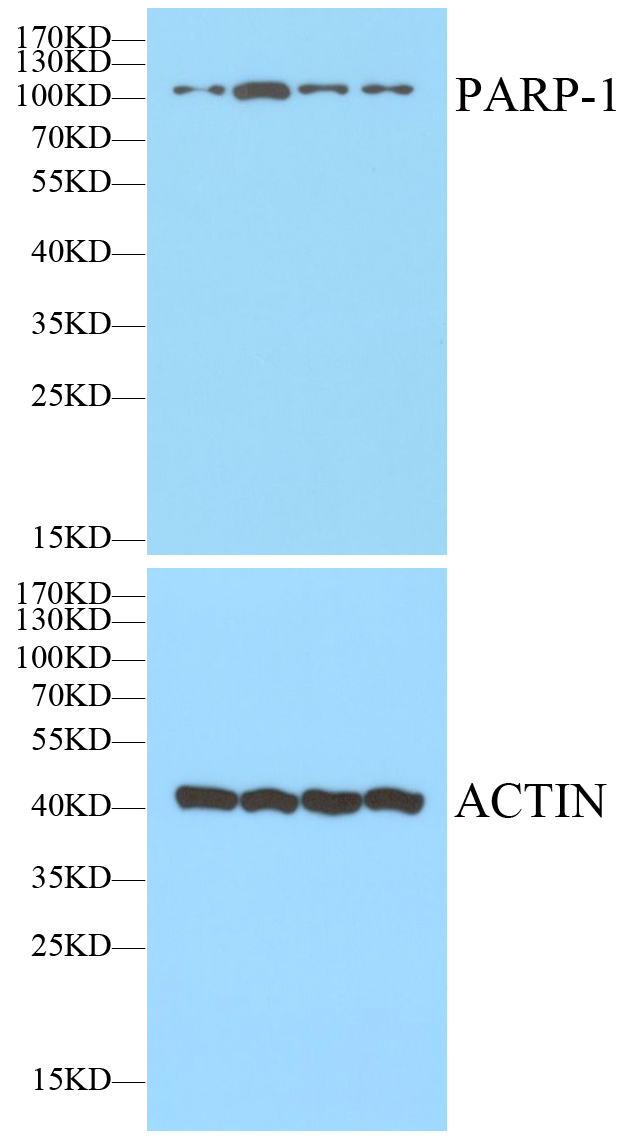


4

3

2

1

The protein levels of PARP-1 in LPS stimulated A549 cells after coculturing with or without mesenchymal stromal cell small extracellular vesicle (MSC sEVs).

1: Sham group

2: LPS+Saline group

3: Sham+sEVs group

4: LPS+sEVs group

**2.** **Figure 6H-PARP-1**

4

3


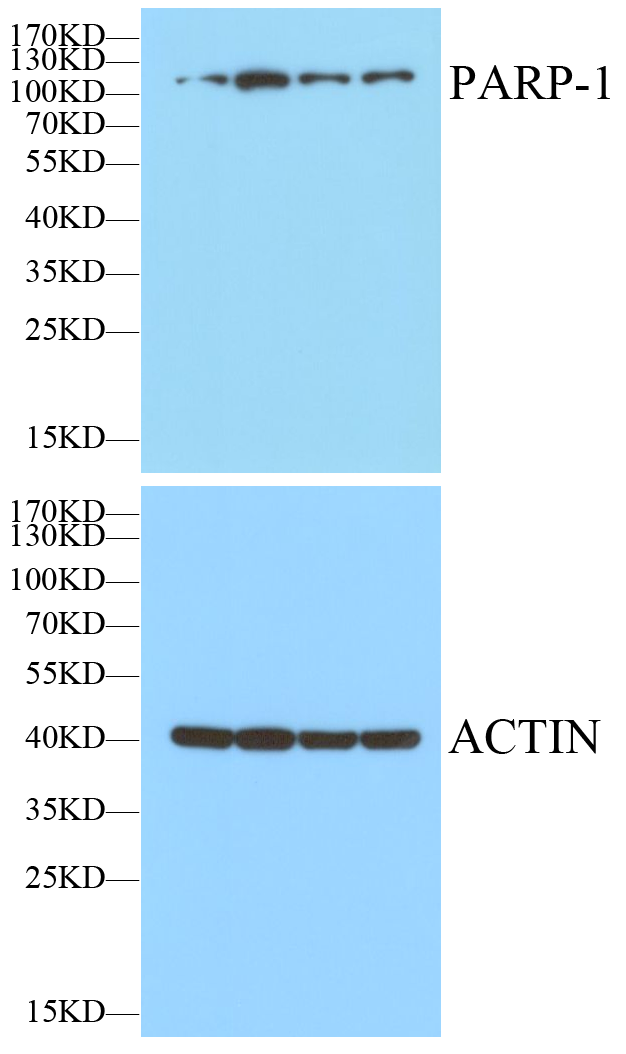


2

1

The protein levels of PARP-1 in LPS stimulated primary human small airway epithelial cells (SAECs) after coculturing with or without mesenchymal stromal cell small extracellular vesicle (MSC sEVs).

1: Sham group

2: LPS+Saline group

3: Sham+sEVs group

4: LPS+sEVs group

**3. Figure 6I-PARP-1**

1

2


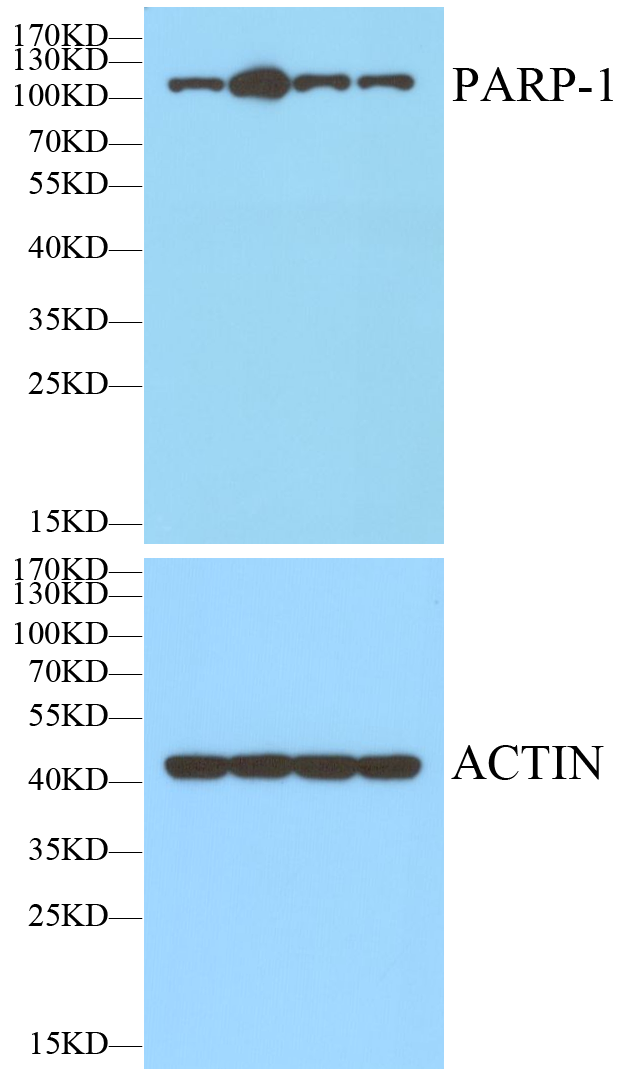


4

3

The protein levels of PARP-1 in lung tissues of LPS mice which were treated with or without mesenchymal stromal cell small extracellular vesicle (MSC sEVs).

1: Sham group

2: LPS+Saline group

3: Sham+sEVs group

4: LPS+sEVs group

**4. Figure 7M-PARP-1**

5

4

3

1

2


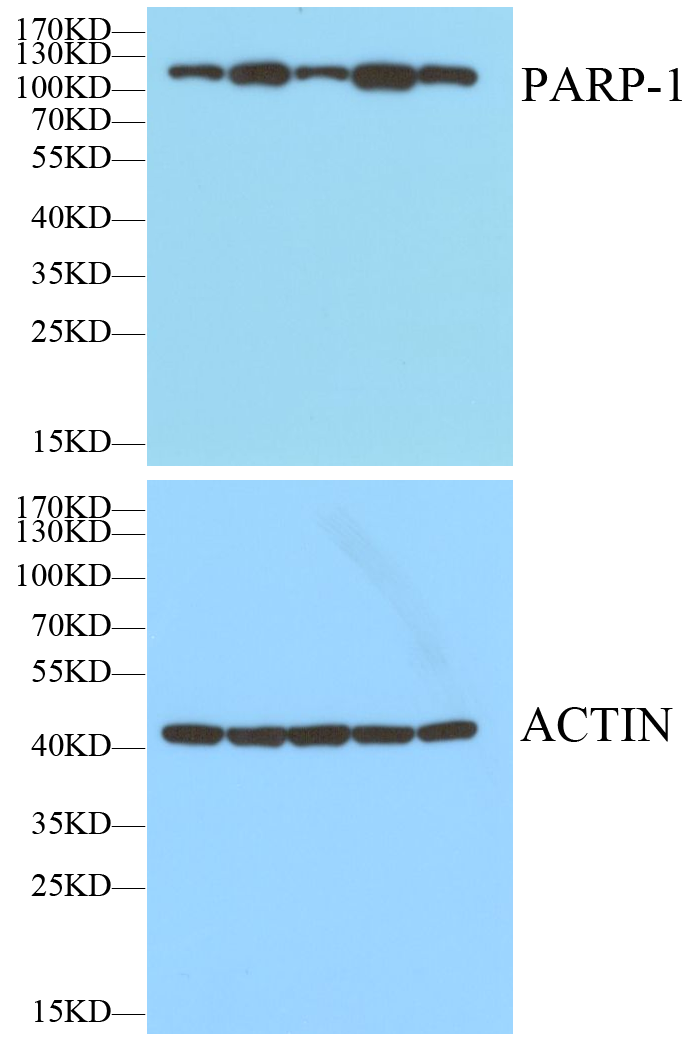


The protein expressions of PARP-1 in A549 cells after coculturing with or without mesenchymal stromal cell small extracellular vesicle (MSC sEVs) which were additionally treated with a specific inhibitor targeting miR-223-3p or control inhibitor.

1: Sham group

2: LPS+Saline group

3: LPS+sEVs group

4: LPS+sEVs (miR-223-3p inhibitor) group

5: LPS+sEVs (control inhibitor) group

**5.** **Figure 7N-PARP-1**


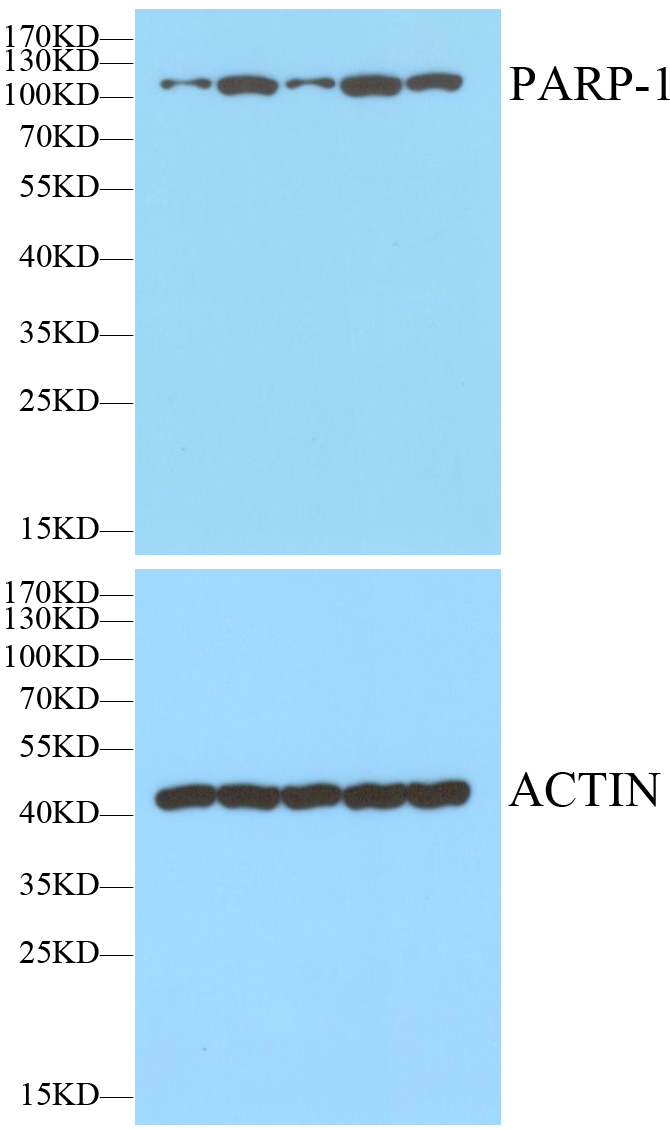


1

2

3

4

5

The protein expressions of PARP-1 in primary human small airway epithelial cells (SAECs) cells after coculturing with or without mesenchymal stromal cell small extracellular vesicle (MSC sEVs) which were additionally treated with a specific inhibitor targeting miR-223-3p or control inhibitor.

1: Sham group

2: LPS+Saline group

3: LPS+sEVs group

4: LPS+sEVs (miR-223-3p inhibitor) group

5: LPS+sEVs (control inhibitor) group

**6. Figure 8H-PARP-1**


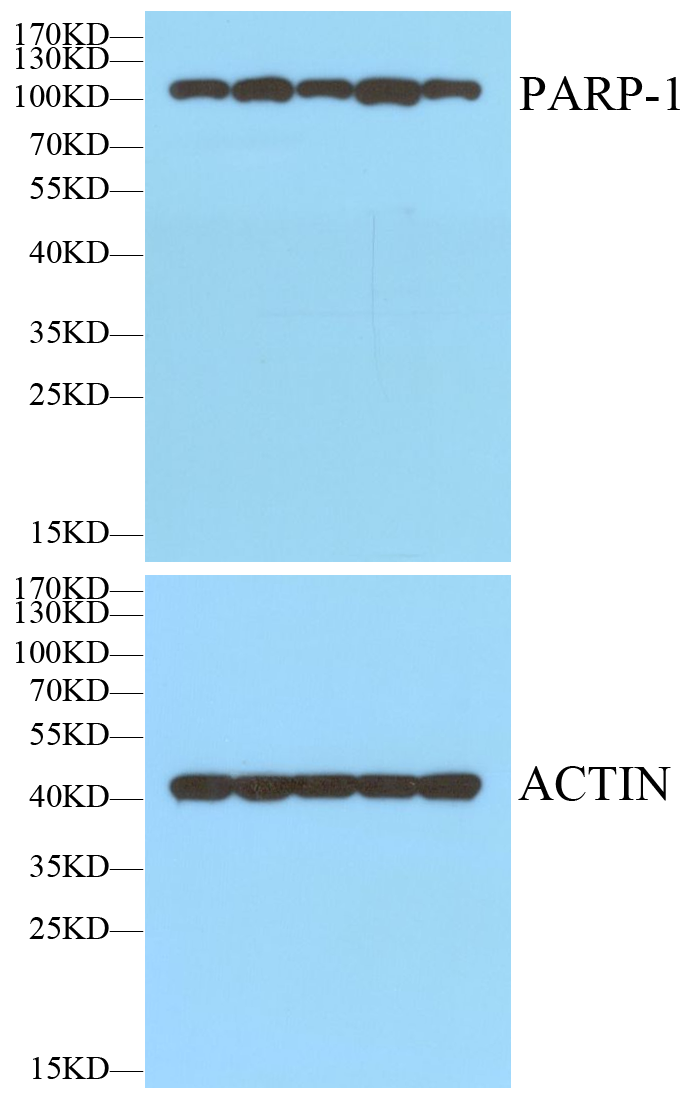


5

4

3

2

1

The protein expressions of PARP-1 in in lung tissues of LPS-induced ALI mice after treatment with or without mesenchymal stromal cell small extracellular vesicle (MSC sEVs) which were additionally treated with a specific inhibitor targeting miR-223-3p or control inhibitor.

1: Sham group

2: LPS+Saline group

3: LPS+sEVs group

4: LPS+sEVs (miR-223-3p inhibitor) group

5: LPS+sEVs (control inhibitor) group

**7. Figure S1B-CD81**


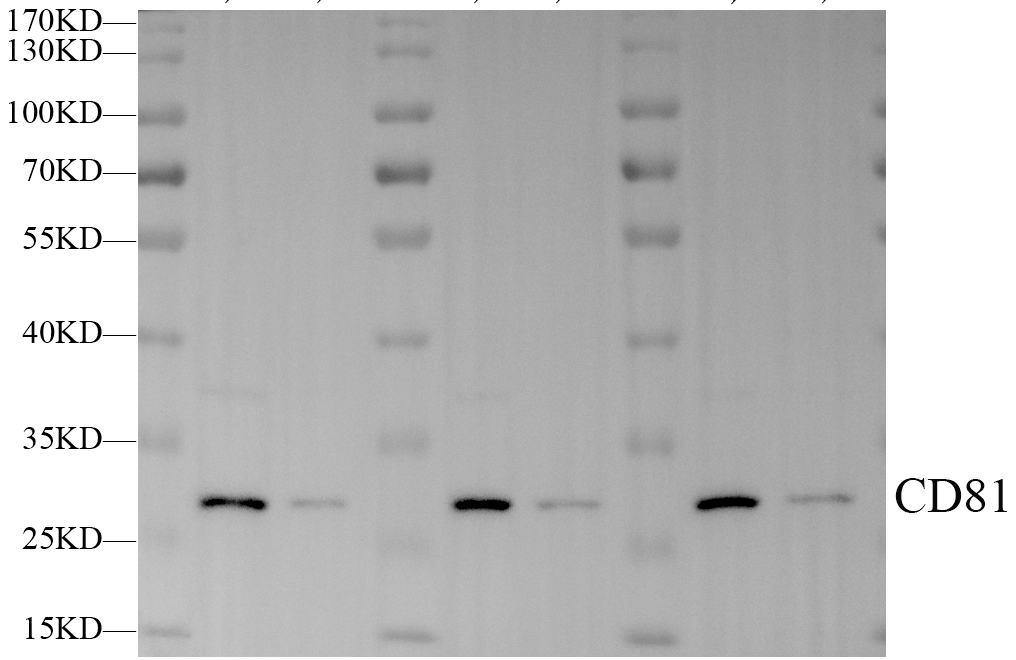


3

1

3

1

2

1

The protein expressions of CD81 in small extracellular vesicle (sEVs) derived from mesenchymal stromal cells (MSCs) and MSCs.

1: MSC sEVs group

2: sEV-depleted conditioned medium group

3: MSC group

**8. Figure S1B-TSG101**


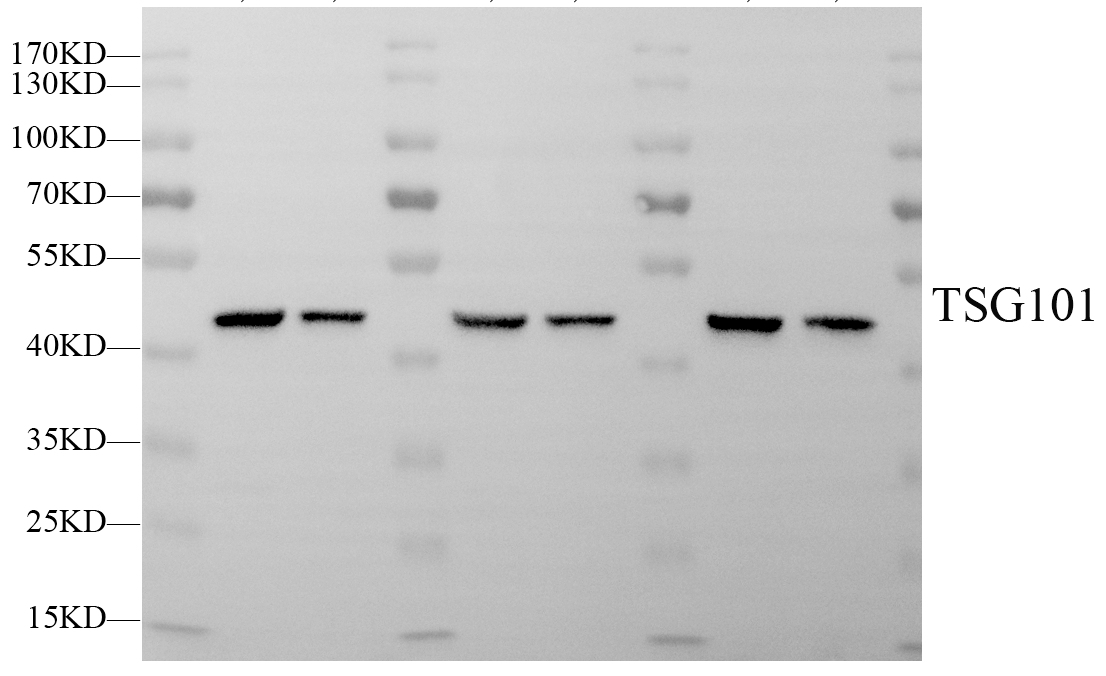


3

1

3

1

2

1

The protein expressions of TSG101 in small extracellular vesicle (sEVs) derived from mesenchymal stromal cells (MSCs) and MSCs.

1: MSC sEVs group

2: sEV-depleted conditioned medium group

3: MSC group

**9. Figure S1B- Calnexin**


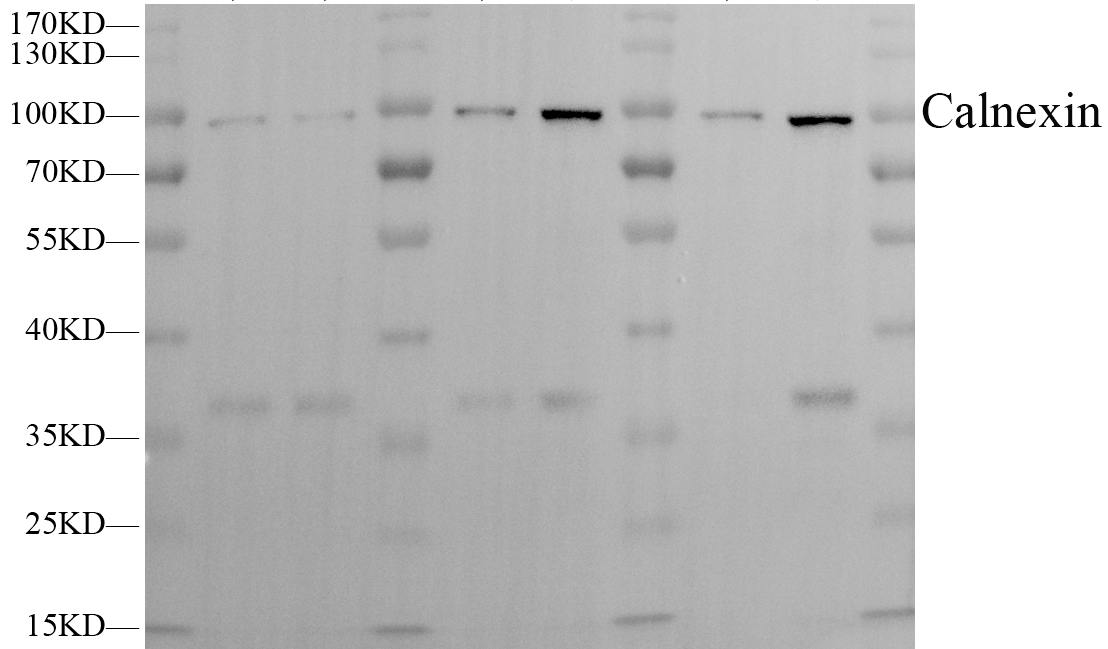


2

1

1

1

3

3

The protein expressions of Calnexin in small extracellular vesicle (sEVs) derived from mesenchymal stromal cells (MSCs) and MSCs.

1: MSC sEVs group

2: sEV-depleted conditioned medium group

3: MSC group
